# Predicting Compressive and Splitting Tensile Strengths of Silica Fume Concrete Using M5P Model Tree Algorithm

**DOI:** 10.3390/ma15155436

**Published:** 2022-08-07

**Authors:** Hammad Ahmed Shah, Moncef L. Nehdi, Muhammad Imtiaz Khan, Usman Akmal, Hisham Alabduljabbar, Abdullah Mohamed, Muhammad Sheraz

**Affiliations:** 1 Department of Civil, Environmental and Ocean Engineering, Stevens Institute of Technology, Hoboken, NJ 07030, USA; 2 School of Civil Engineering, Central South University, Changsha 410075, China; 3 Department of Civil Engineering, McMaster University, 1280 Main Street West, Hamilton, ON L8S 4L7, Canada; 4 Department of Civil Engineering, University of Engineering & Technology Peshawar, Bannu 28100, Pakistan; 5 Department of Civil Engineering, University of Engineering and Technology, Lahore 54890, Pakistan; 6 Department of Civil Engineering, College of Engineering in Al-Kharj, Prince Sattam Bin Abdulaziz University, Al-Kharj 11942, Saudi Arabia; 7 Research Centre, Future University in Egypt, New Cairo 11832, Egypt

**Keywords:** concrete, silica fume, compressive strength, splitting tensile strength, artificial intelligence, model, M5P tree algorithm

## Abstract

Compressive strength (CS) and splitting tensile strength (STS) are paramount parameters in the design of reinforced concrete structures and are required by pertinent standard provisions. Robust prediction models for these properties can save time and cost by reducing the number of laboratory trial batches and experiments needed to generate suitable design data. Silica fume (SF) is often used in concrete owing to its substantial enhancements of the engineering properties of concrete and its environmental benefits. In the present study, the M5P model tree algorithm was used to develop models for the prediction of the CS and STS of concrete incorporating SF. Accordingly, large databases comprising 796 data points for CS and 156 data records for STS were compiled from peer-reviewed published literature. The predictions of the M5P models were compared with linear regression analysis and gene expression programming. Different statistical metrics, including the coefficient of determination, correlation coefficient, root mean squared error, mean absolute error, relative squared error, and discrepancy ratio, were deployed to appraise the performance of the developed models. Moreover, parametric analysis was carried out to investigate the influence of different input parameters, such as the SF content, water-to-binder ratio, and age of the specimen, on the CS and STS. The trained models offer a rapid and accurate tool that can assist the designer in the effective proportioning of silica fume concrete.

## 1. Introduction

Concrete has been the world’s most used construction material for many decades owing to its mechanical properties, durability, availability of ingredients, and versatility. It is estimated that in 2018, approximately 4.1 billion tons of ordinary Portland cement was produced worldwide, with an annual growth of approximately 5% [1]. However, the cement industry is considered one of the primary contributors to anthropogenic CO_2_ emissions. Approximately 7% of global CO_2_ emissions emanate from the construction industry [1]. Due to the alarming threat of climate change, sustainable concrete with enhanced mechanical properties has been developed, incorporating many chemical admixtures and pozzolanic materials such as silica fume (SF).

**Figure 1:**
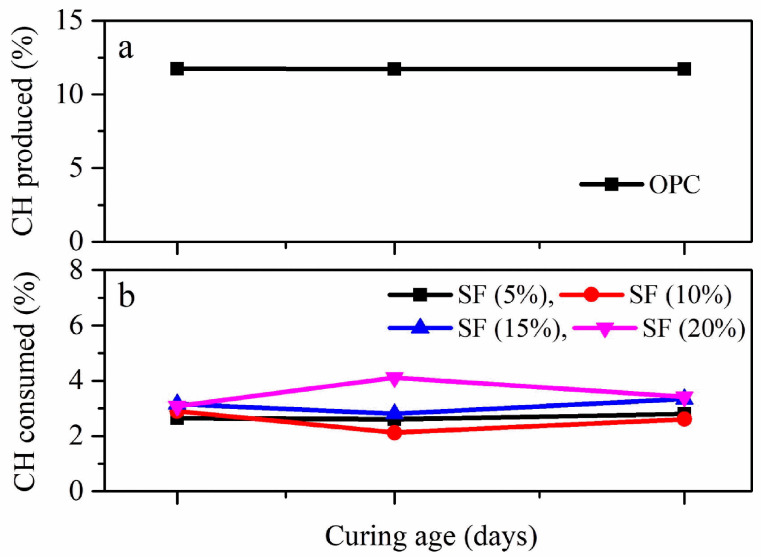
The amount of CH produced by (**a**) OPC and (**b**) consumed by silica fume at different percentages of cement replacement [6].

SF is a by-product of electric arc furnaces used to make ferrosilicon alloys and silicon. SF, also known as microsilica, is composed of ultrafine particles with a surface area of approximately 20,000 m^2^/kg and an average particle size that is almost 100 times smaller than that of cement particles. Due to the fact of its fineness, it is a highly effective pozzolanic material with a reasonably quick reactivity in addition to its exceptional micro-filling capacity [2]. From small dosages to up to 50%, SF is added to concrete. However, research shows that the most advantageous and useful dosage range is 5–20%. Its predominant finer fraction acts as a pozzolanic material and reacts in the early stages of strength development, yet it may include larger particles that could persist unreactive at later stages [3]. During cement hydration, C_3_S and C_2_S in cement particles are hydrated and form calcium silicate hydrate (C-S-H) gel, which plays a vital role in the mechanical strength of concrete. Moreover, some amounts of Ca(OH)_2_ are produced, remain unreactive, and do not contribute significantly to strength enhancement. With the addition of SF, amorphous silica (SiO_2_) reacts with the Ca(OH)_2_ and produces an additional amount of C-S-H gel, which contributes to mechanical strength enhancement, denser microstructure, and more durable concrete [4]. Studies show that the addition of SF significantly decreases the amount of calcium hydroxide at 3 days. According to the literature, regardless of the water-to-cement (w/c) ratio, all of the Ca(OH)_2_ was used when 16% SF partial replacement for cement was used [5]. Figure 1b depicts that the amount of CH consumed by the 15% and 20% SF dosages was higher compared with the dosage of 5% at 7, 28, and 56 days [6]. Moreover, it was reported that 12% SF and 0.8% nanosilica increased the total heat of hydration by 48.49% compared to ordinary concrete, while the porosity of the concrete decreased by 6.14%, which resulted in a denser microstructure [7]. Due to the presence of these benefits, SF is also used in ultra-high-performance concrete [8]. However, care should be taken while using SF in concrete with air entrainment, because it may increase the dosage of air-entraining admixtures required for certain air content [9]. 

The addition of SF as a partial replacement for cement increases the compressive strength (CS) by improving the density and strength of its cement paste constituent and densifying the cement paste aggerate transition zone. It also enhances the homogeneity of concrete and decreases the number of large pores. Concrete incorporating SF is improved in two primary ways. First, SF decreases the porosity owing to its excellent filling property. It also increases the bond strength between the hydrated cement paste and aggregates owing to the additional amount of C-S-H gel [10] produced by the pozzolanic reaction, consuming the large plates of portlandite that constitute a weak link, and densifying the interfacial zone through the powerful microfiller effect of the spherical and very fine SF particles. The resulting enhancement of CS and other properties depends on the dosage of SF and the w/c ratio. SF can typically improve the CS of concrete by 6–57%. It has also been observed that with the addition of 5%, 10%, and 15% SF, the splitting tensile strength of concrete was enhanced by 9.7%, 54%, and 85.9%, respectively [11]. Different studies concluded that if other mixture design parameters are kept constant, the optimum content of SF for 28 days of strength is a function of the w/c ratio. At 15% SF and a w/c ratio of 0.26, the values of the CS and the splitting tensile strength (STS) at 28 d were 95.7 MPa and 6.65 MPa, respectively [12,13]. Moreover, it was noted that when SF was used in a mixture incorporating steel fibers, both the CS and STS increased, enhancing the bond strength between the fibers and the matrix [11]. In addition to SF, other materials such as cellulose nanocrystals and carbon nanotubes can be used to enhance the tensile property of composite materials [14,15]. 

There is a growing trend toward the use of supplemental cementitious materials (e.g., fly ash, slag, calcinated clays, rice husk ash, and silica fume) in a concrete structure in order to achieve sustainability and ecoefficiency. Due to the highly non-homogeneous nature of the mixture and the nonlinear relationships between the components of the mixture and the engineering properties, the proportioning of concrete’s mixture, the choice of the optimal dosages of its constituents, and the prediction of the resulting engineering properties, such as CS and STS, present a recurrently complex problem. For example, SF has frequently been utilized in concrete to achieve a number of performances and sustainability goals. However, there is still a continuing need for the development of trustworthy and reliable models that can accurately predict the CS and STS of silica fume concrete without the need for expensive and time-consuming laboratory trial batches and experimental tests.

A combination of appropriate input parameters is needed for desirable mechanical properties, and there is a need to perform many experimental studies to determine this combination. Appropriate predictive models can reduce these attempts and prevent the material waste associated with the conventional mixture proportioning method. Consequently, productive models can save time and cost and accelerate construction schedules and activities such as formwork removal or prestressing. For many years, researchers have been using machine learning (ML) techniques for the prediction of different properties of sustainable concrete owing to their superior accuracy and robustness [16]. For example, Hammad et al. [17], Ahmad et al. [18], Rajeshwari et al. [19], Mohammed et al. [1], and Song et al. [20] predicted the mechanical strength of concrete with fly ash by using different ML techniques. Similarly, ML techniques were employed for estimating the mechanical properties of concrete with sustainable materials such as ground granulated blast furnace slag [21], metakaolin [22], rice husk ash [23], and recycled aggregate [24]. Nonetheless, a dearth of studies have explored modeling the CS and STS of concrete incorporating SF using ML techniques that provide a simple mathematical equation for practical purposes. To fill this research gap, the M5P model tree algorithm was used in this study for the prediction of the CS and STS of concrete made with SF. The M5P model tree algorithm has been used for predicting different properties of cement-based materials [16]. Accordingly, a large database was compiled from peer-reviewed published documents. The performance of the developed M5P models was compared with that of linear regression analysis and gene expression programming. Moreover, parametric analyses were carried out to investigate the variation in the CS and STS of concrete with different input parameters such as the SF content, water-to-binder (w/b) ratio, and age of the specimens.

## 2. Data Collection

**Figure 2:**
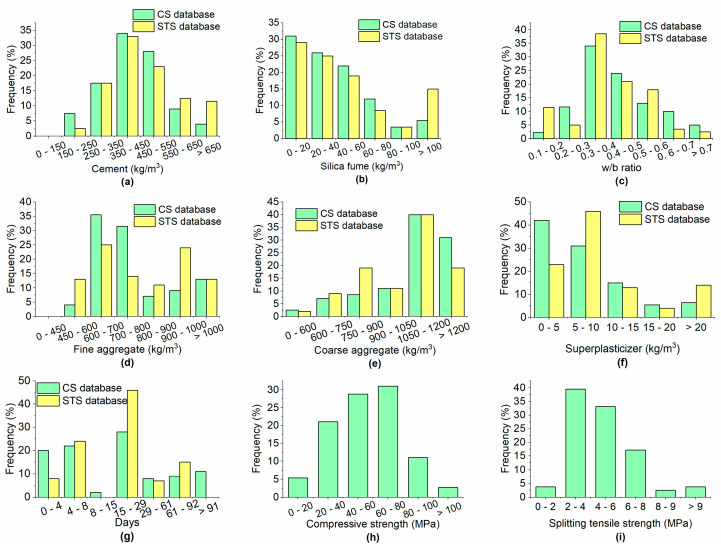
Histograms of (**a**) cement; (**b**) SF; (**c**) w/b ratio; (**d**) FA; (**e**) CA; (**f**) SP; (**g**) days; (**h**) CS; (**i**) STS.

The databases for this paper were compiled from previously published literature [2,3,10,11,25,26,27,28,29,30,31,32,33,34,35,36,37,38,39,40,41,42,43,44,45,46,47,48,49,50,51,52,53,54,55]. The database for CS consisted primarily of data examples on cubic specimens, while data obtained on cylindrical specimens were converted into cubic specimens using suitable factors widely adopted in the literature. The CS of cylindrical specimens of the dimensions ∅100 mm×200 mm was converted into the CS of a cube with dimensions of 150 mm, by multiplying by a factor of 0.98 for high-strength concrete (HSC) (the definition of HSC can be found in ACI 363R) and by 1.1 for normal-strength concrete (NSC). Moreover, the factors 0.9 and 0.96 were used to convert the CS of 100 mm cubes into the CS of 150 mm cubes for HSC and NSC, respectively [56]. The database for STS mainly consisted of data obtained on cylindrical specimens. The results of the STS obtained from cylinders of ∅150 mm ×300 mm did not change, while the STS results of cylinders of ∅100 mm ×200 mm were converted into the STS of cylinders of ∅150 mm ×300 mm by multiplying by a factor of 0.91 [57,58]. For modeling the CS of concrete with SF, 796 data examples were used, while the database for STS was composed of 156 data points. The database for CS can be found in Supplementary Materials (Table S1) as well as the database for STS (Table S2). Each input example included the cement (C), SF, fine aggregate (FA), coarse aggregate (CA), and superplasticizer (SP) dosages, along with the age of the specimen in days, while the CS and STS were the output parameters. The histograms of the variables for both the CS and STS databases are shown in Figure 2. The descriptive statistics of the input and output parameters for the dataset used in the training set are given in Table 1.

## 3. Methodology

### 3.1. M5P Model Tree Algorithm

The M5 algorithm was originally proposed by Quinlan [59], while the M5P algorithm [60] is an expanded form of the original M5 algorithm. The M5P algorithm was modified from M5 to deal with enumerated attributes and attributes with missing values.

**Figure 3:**
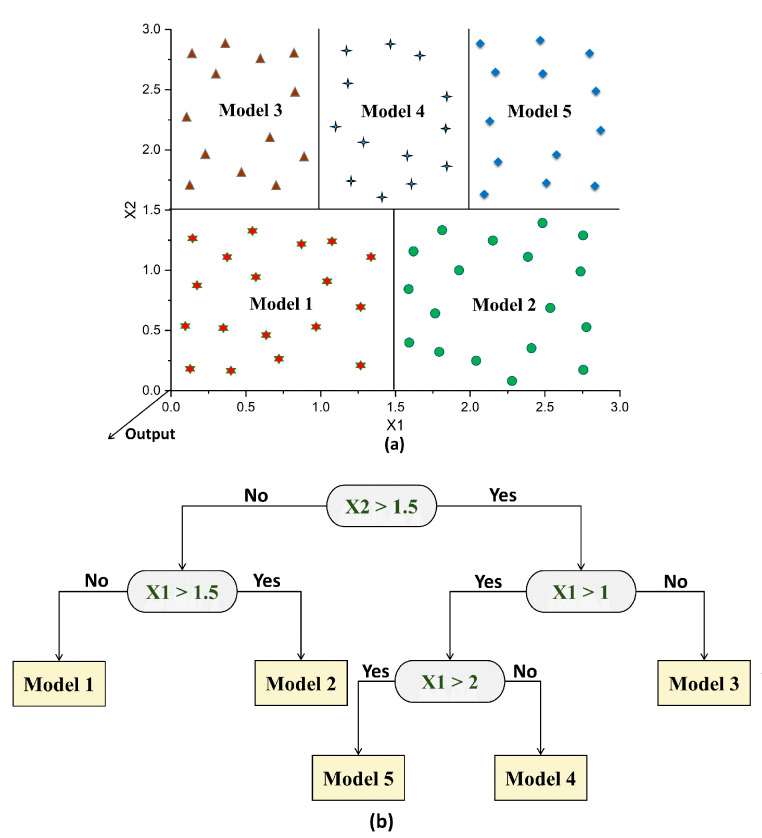
Illustration of the M5 algorithm: (**a**) splitting of the input space; (**b**) building of the tree.

An illustration of the M5 algorithm is depicted in Figure 3. The input data are split into several subspaces, and each subspace comprises data with shared features (Figure 3a). Within a particular subspace, linear regression models are used to reduce the variation in the data. Then, several nodes are created based on information obtained from the previous step in which the splitting process is carried out based on a given attribute (Figure 3b). This step permits the construction of an inverted tree-like structure.

The M5P tree model algorithm comprises four main steps. In the beginning, the input space is divided into various subspaces that form a tree. A splitting criterion is used to reduce the intra-subspace irregularity down from the root to a node. At the node, the standard deviation of values is used to compute the variability. In order to minimize possible errors at the node and help to build a tree, a standard deviation reduction (*SDR*) is used as follows:

(1) $$SDR=sd\left(S\right)-\sum_{i}\frac{S_{i}}{\left|S\right|}\times sd\left(S_{i}\right)$$

where sd is the standard deviation; S is the dataset that comes to the node; $S_{i}$ are the sets produced by the splitting node in accordance with a given attribute [60]. After constructing the tree, the second stage involves creating a linear regression model in each of the subspaces using the data from that subspace. An over-training issue arises when the linear model’s *SDR* at the sub-root tree is lower than the predicted error for the sub-tree. A pruning strategy is used to manage the overtraining issue. The pruning procedure, however, might lead to abrupt discontinuities among adjacent linear models. The smoothing procedure is completed in the last stage to solve this issue. By combining all models from the leaf to the root, the final model of the leaf is created during the smoothing phase. The estimated value of the leaf is subsequently filtered.

### 3.2. Comparison of the M5P Models with Other Modeling Techniques

To evaluate the capability of the proposed M5P models in the prediction of the CS and STS of concrete incorporating SF, they were compared with linear regression analysis and gene expression programming (GEP). These techniques used for comparison purposes have often been deployed in modeling different properties of cement-based materials [16]. The mathematical equation for linear regression is given in Equation (2).

(2) $$Y=a+\sum_{i=1}^{k}\beta_{i}x_{i}$$

where Y is an output parameter; a is a constant; $\beta_{i}$ are the coefficients; $x_{i}$ (*i =* 1, 2, …, *k*) are the inputs parameters.

GEP is a branch of AI that was developed by Ferreira [61]. It comprises five components that can be divided into two categories based on their functions. The search space of the algorithm is controlled by the function set, terminal set, and fitness function, while its quality and speed of search are regulated by control parameters and terminal conditions. Due to the multigenic nature of GEP, complex and nonlinear programs with different subprograms were developed. More details regarding the methodology of GEP can be found in [61].

## 4. Model Development and Evaluation Criteria

For both databases of CS and STS, 67% of the data was used for the training set and 33% was used for the testing set as suggested by Hammad et al. [17]. The M5P model was applied using the Waikato Environment for Knowledge Analysis (WEKA) software. The M5P algorithm generates linear regression mathematical equations after making different classes of data. The general form of the M5P algorithm can be written as follows:

(3) $${f^{\prime }}_{c},\;f_{\mathrm{st}}=a+\left(b\times C\right)+\left(c\times \mathrm{SF}\right)+\left(d\times w/b\right)+\left(e\times \mathrm{FA}\right)+\left(f\times \mathrm{CA}\right)+\left(g\times \mathrm{SP}\right)+\left(h\times \mathrm{days}\right)$$

For model tree development, the minimum number of instances for CS was kept at 40 in order to keep a balance between the number of developed linear models and accuracy in terms of a higher value of a correlation coefficient (*R*) and coefficient of determination (*R*^2^). For developing the M5P model for STS, all the settings of the software were kept at default. The default settings were selected because they attained the best performance in terms of the high value of *R*^2^ and the low value of the root mean square error (*RMSE*).

For developing GEP models, the GeneXproTools 5.0 software was used. For both the CS and STS databases, several GEP models were developed by changing the parameters of the algorithm. The parameters of the best GEP models are given in Table 2. It should be noted that the accuracy of the GEP models in both the training and testing sets changed by varying the head size, chromosome, genes, linking function, and number of generations, as studied by Hammad et al. [17]. All other parameters were kept at their respective default values because of the high performance of the developed models at the default settings observed after multiple trial and error tests.

Different statistical metrics were used to evaluate the performance of the developed models including *R*, *R*^2^, mean absolute error (*MAE*), relative squared error (*RSE*), *RMSE*, and discrepancy ratio (*DR*). The mathematical formulations of these statistical metrics are given in Equations (4)–(9) below.

(4) $$R=\frac{\sum_{i=1}^{k}\left(e_{i}-{\bar{e}}_{i}\right)\left(p_{i}-{\bar{p}}_{i}\right)}{\sqrt{\sum_{i=1}^{k}{\left(e_{i}-{\bar{e}}_{i}\right)}^{2}\sum_{i=1}^{k}{\left(p_{i}-{\bar{p}}_{i}\right)}^{2}}}$$

(5) $$R^{2}\;=\;1-\;\left(\frac{\sum_{i=1}^{n}{\left(e_{i}-p_{i}\right)}^{2}}{\sum_{i=1}^{n}{\left(p_{i}\right)}^{2}}\right)$$

(6) $$MAE=\frac{\sum_{i=1}^{n}\left|e_{i}-p_{i}\right|}{n}$$

(7) $$RSE=\frac{\sum_{i=1}^{n}{\left(p_{i}-e_{i}\right)}^{2}}{\sum_{i=1}^{n}{\left(\bar{e}-e_{i}\right)}^{2}}$$

(8) $$RMSE=\sqrt{\frac{1}{n}\sum_{i=1}^{n}{\left(e_{i}-p_{i}\right)}^{2}}$$

where $e_{i}$ and $p_{i}$ are the experimental and predicted values, respectively; $\bar{e}$ is the average experimental value; n represents the total number of samples.

A model with *R*^2^ < 0.7 indicates poor performance [62], while a model with *R* > 0.8 generally shows a strong positive correlation between the model estimated and experimental results [63]. The *RSME*, *MAE*, and *RSE* capture the accuracy of the proposed model; higher values of these statistical metrics indicate that the model’s predicted results are far from the actual experimental results, while lower values insinuate that the model’s estimated results have acceptable accuracy. When *DR* = 0, this shows that the actual and estimated results exactly match each other, while negative and positive *DR* values indicate underestimation and overestimation, respectively [17]. In this study, for both the CS and STS databases, the accuracy was defined by values of *DR* ranging from −0.1 to 0.1.

## 5. Results and Discussion

### 5.1. Compressive Strength

**Figure 4:**
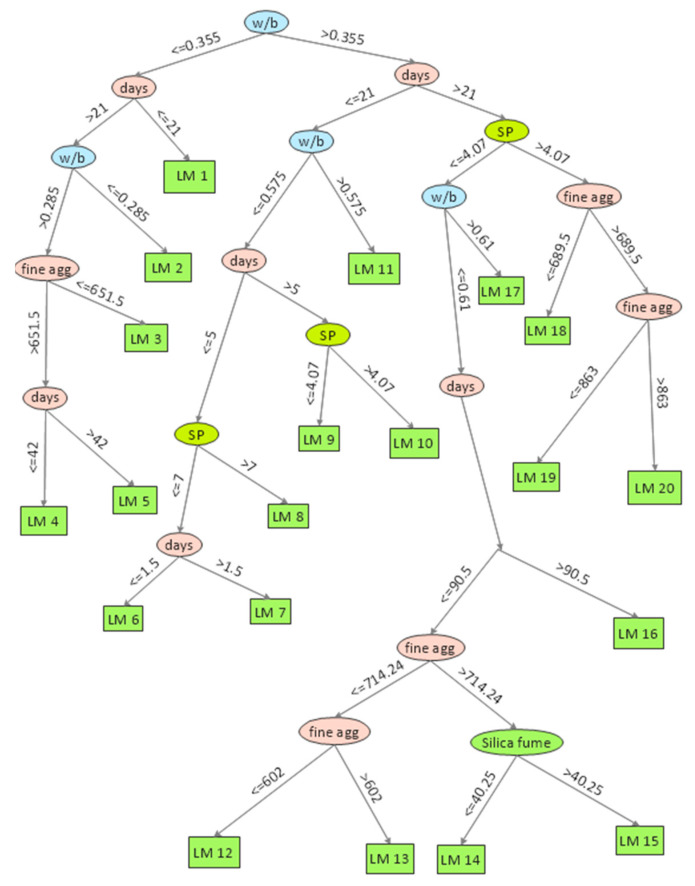
Generated model tree structure of M5P for CS.

**Figure 5:**
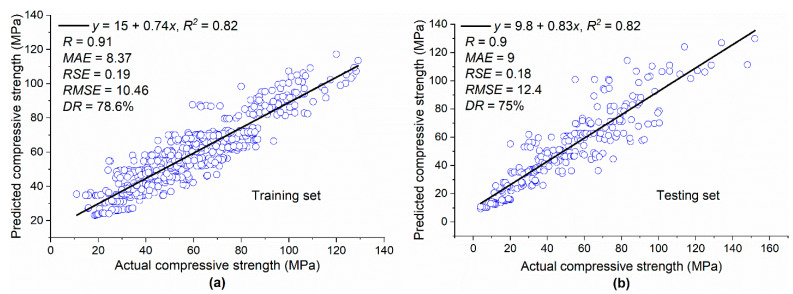
Actual and predicted values of CS: (**a**) training set; (**b**) testing set.

Based on Equation (3), model trees were generated as shown in Figure 4. The term LM at the tree leaves represents the linear model identified by the M5P algorithm. The corresponding coefficients for linear models developed by M5P for CS based on Equation (4) are given in Table 3. Figure 5a,b depict a comparison between the actual results and the model’s predicted values for both the training and testing datasets for the CS database. For both the training and testing sets, the value of *R*^2^ was 0.82, which shows that the M5P model was well trained based on the training set and gave prediction results with high accuracy for new data unfamiliar to the model and thus far unseen in the testing set. Moreover, the high values of *R* and *DR* and low values of *MAE*, *RSE*, and *RMSE* for both datasets confirmed that the M5P model predicted the CS of silica fume concrete with high accuracy. One of the advantages of M5P over other machine learning techniques, such as gene expression programming, is that it gives a simple linear mathematical equation for predicting the desired property [17] and is not a mere backbox tool.

**Figure 6:**
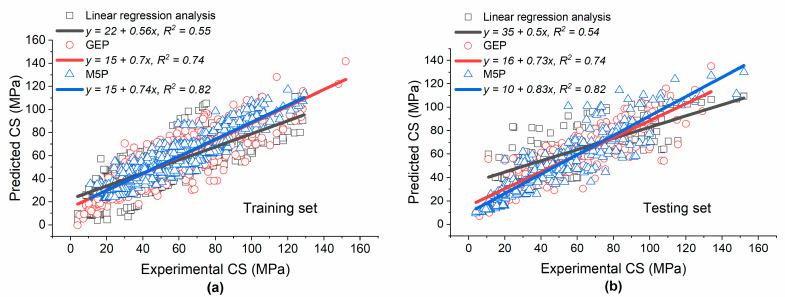
Comparison of the linear regression analysis, GEP, and M5P for the prediction of the CS of concrete made with SF: (**a**) training dataset; (**b**) testing dataset.

Figure 6 depicts a comparison between the performance of the linear regression analysis, GEP, and M5P in predicting the CS of concrete made with SF. It can be observed that for both the training and testing datasets, M5P had the highest value of *R*^2^ followed by GEP and linear regression analysis, respectively. Although the accuracy of linear regression analysis was lower compared with that of the other modeling techniques, its primary advantage is that it gives one simple mathematical equation for estimating the CS. In the case of GEP, the accuracy was lower compared to that of M5P. M5P provides simple mathematical equations for calculating CS, while GEP often generates complex nonlinear empirical equations, which may be inconvenient to use [17]. The higher performance of M5P compared with the other modeling techniques considered herein is further confirmed in Table 4, which shows that the values of *R* and *DR* for M5P were higher compared with the corresponding values for GEP and the linear regression analysis, while the values of *RSE*, *RMSE*, and *MAE* for M5P were lower.

### 5.2. Splitting Tensile Strength

**Figure 7:**
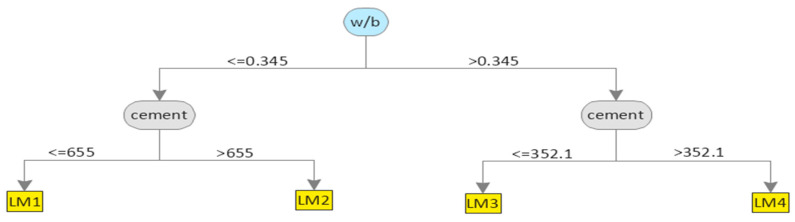
Generated model of the tree structure of M5P for STS.

**Figure 8:**
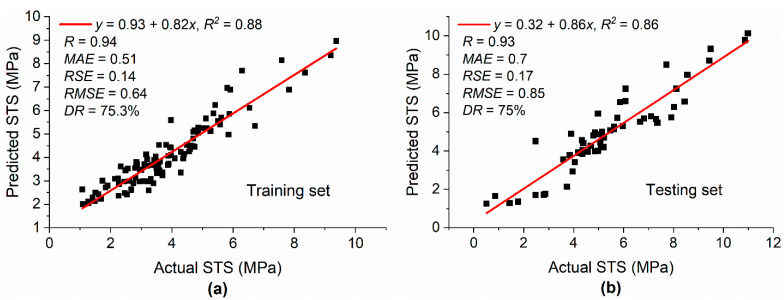
Actual and predicted values of STS: (**a**) training set; (**b**) testing set.

The model tree structure generated by M5P for STS is depicted in Figure 7, and the coefficients of the developed linear models are given in Table 5. The values of *R*^2^ were 0.88 and 0.86 for the training and testing datasets, respectively, as shown in Figure 8, which was slightly higher compared with the corresponding values for the M5P model developed for CS. In Figure 8, the slope of the regression line for both datasets was close to 1, showing that the M5P model was well trained and captured the relationship between input and output variables, which allowed for predicting the STS of silica fume concrete with high accuracy. Moreover, the high value of *R* and low value of *RSE* for both the training and testing datasets indicate that the difference between the actual and model estimated results were low and that the predicted and estimated values were close to each other.

**Figure 9:**
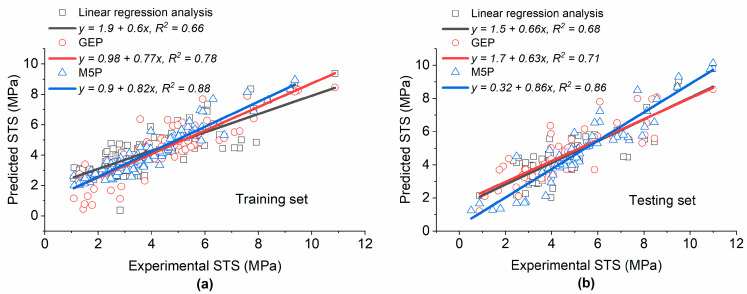
Comparison of the linear regression analysis, GEP, and M5P for the prediction of STS of concrete made with SF: (**a**) training set; (**b**) testing set.

A comparison of the different modeling techniques used for the estimation of the STS of concrete with SF is presented in Figure 9. For the training set, the accuracy of M5P was superior (*R*^2^ = 0.88) compared with that of GEP (*R*^2^ = 0.78) and the linear regression analysis (*R*^2^ = 0.66). A similar trend was observed for the testing set. The value of the slope of the regression line (0.82 and 0.86 for the training and testing set, respectively) was close to 1, which indicates that the difference between the actual and predicted values was low. The higher performance of M5P over GEP and the linear regression analysis is also displayed in Table 6, which shows that the value of *R* for M5P was higher for both datasets, while the values of *RMSE*, *RSE*, and *MAE* were lower. Although different statistical metrics show that the performance of GEP was higher compared with that of the linear regression analysis, the value of *DR* in the testing set for the former was lower compared to the latter. Therefore, it would be better to use a variety of statistical metrics for the comparison of different models instead of relying only on limited metrics.

### 5.3. Parametric Analysis

Dedicated parametric analysis (PA) was carried out to determine the effect of variation in the output parameters by changing the input parameters from their respective minimum values to maximum values. The output parameters were the CS and STS, while the input parameters included the w/b ratio, age of concrete in days, and SF dosage. The input parameters were changed one at a time while maintaining the other parameters at their mean value and recording the corresponding change in the output parameters.

**Figure 10:**
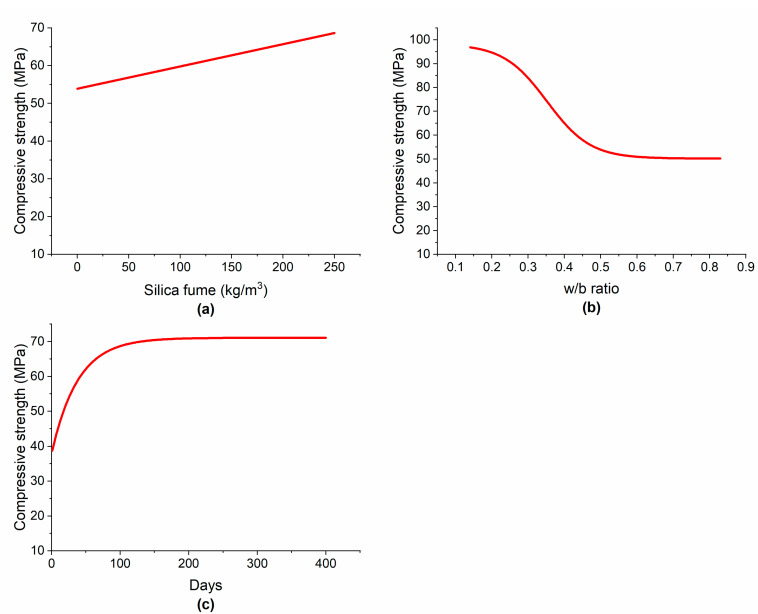
Variation in the CS by changing: (**a**) silica fume; (**b**) w/b ratio; (**c**) curing time.

Figure 10a shows that by increasing the SF content from 0 to 250 kg/m^3^, the CS increased linearly from approximately 54 to 69 MPa. This increase in strength can be attributed to the pozzolanic activity of SF and its capacity for microfilling because of its ultrafine particles, which increase the density of the matrix and the transition zone between the cement paste and aggregate, densifying the microstructure and strengthening the bond between the paste and aggregate [12]. When the SF is added to the concrete, it is claimed that the interfacial transition zone (ITZ) between the paste and aggregates is less porous and has a homogenous microstructure [64]. It was reported that the reduction in ITZ was 25% by adding 10% SF, while the reduction was 65% at 30% SF [65]. It was illustrated that by incorporating 6% and 12% SF, porous, rough, and heterogeneous concrete microstructural characteristics transformed into dense, flat, and homogeneous features, respectively [66]. Table 7 indicates that the use of SF in concrete decreased the porosity, consequently increasing CS. Similar to the present results, several researchers observed an increase in CS with the incorporation of SF [10,30]. However, it was also reported that beyond a certain threshold, a higher SF dosage was not beneficial for CS enhancement. For example, Siddique et al. [12] noted that SF enhanced the CS of concrete; however, at a very high content, it was not effective for an increase in CS, yet it was effective at improving other hardened properties such as the flexural strength. Due to the fact of its large surface area, a higher dosage of SF absorbs more water, and more SP is needed to make the mixture workable, which also increases the cost [10].

**Figure 11:**
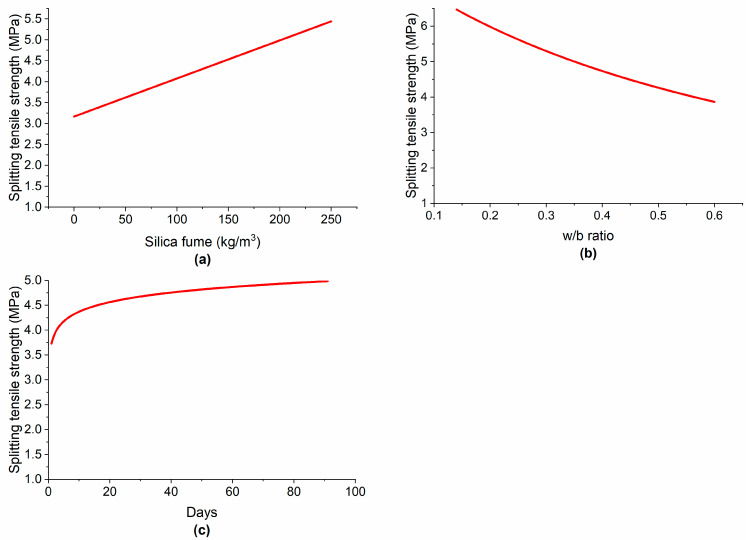
Variation in STS with: (**a**) silica fume; (**b**) w/b ratio; (**c**) curing time.

The STS of concrete also increased linearly by increasing the SF content, as shown in Figure 11a. Mazloom et al. [10] studied the effect of SF on the hardened properties of concrete and found that increasing the SF content in concrete enhanced the modulus of elasticity and increased the STS of concrete. However, these results do not concur with the findings of Hooton et al. [68], who posited that increasing the SF content decreased the tensile strength of concrete. Similar to CS, the SF content has shown a linear relationship with STS. This is because there exists a positive correlation between the CS and STS of concrete incorporating SF. Bhanja et al. [13] tested 32 concrete mixtures made with different w/c ratios and SF dosages and found an empirical relationship between the CS and STS of concrete and the SF dosage.

The influence of the w/b ratio on the CS is illustrated in Figure 10b. By increasing the w/b ratio from 0.15 to 0.55, the CS decreased from approximately 97 to 52 MPa (−46%). This is due to the well-known fact that increasing the w/c ratio creates additional voids and increases the porosity, thus decreasing the density of concrete and, ultimately, leading to CS reduction [4]. By further increasing the w/b ratio from 0.55 onward, only a slight reduction in CS was observed. In the case of STS, by increasing the w/b ratio from approximately 0.15 to 0.6, the STS decreased by approximately 41% as shown in Figure 11b.

Concrete gains its mechanical strength gradually with curing time and age, depending on many factors including the type of binder and other ingredients used, the mixture proportions, and curing conditions. In the early stages, concrete gains its strength very quickly due to the rapid hydration reactions. Figure 10c shows that the increase in CS in the first 28 days was almost linear. This may be attributed to the pozzolanic reactions of SF with the calcium hydroxide from cement hydration. By the further passage of time up to about 90 days, the CS increased nonlinearly. After 90 days and onward, the advancement in CS of concrete did not stop but continued at a very slow rate. Siddique et al. [12] stated that beyond 90 days, concrete gained compressive negligibly because the inhibiting layers of hydration reactions materials prevent SF from reacting with calcium hydroxide so the increase in CS became very low. After approximately 120 days, no significant change in CS was observed. Similar to the present results, Hooton [68] concluded that long-term strength gain in concrete made with SF was very low. Figure 11c indicates that concrete gains STS almost linearly in the first 7 days. At 28 days, concrete incorporating SF gains most of its STS. Siddique et al. [12] also noted that the tensile strength of concrete increased mostly in the first 28 days. After that, there was little enhancement in tensile strength.

## 6. Conclusions

In many codes, the design parameters for concrete are its compressive strength (CS) and splitting tensile strength (STS). The cost and time can be reduced in addition to scheduling tasks such as the removal of formwork with a precise and reliable prediction of these characteristics. In this study, silica fume (SF) was used as a partial cement replacement and the CS and STS of concrete with SF were modeled using M5P and compared with linear regression analysis and gene expression programming (GEP). Thorough databases were developed for this purpose from peer-reviewed published materials. The information utilized in the modeling contained 156 data points for STS and 796 data points for CS of concrete with SF. Cement, SF, w/b, fine and coarse aggregates, superplasticizer, and specimen age in days were the input parameters for both databases. To assess the predictive abilities of M5P, linear regression analysis, and GEP, numerous statistical indicators were employed. Finally, parametric analysis (PA) was carried out. The following conclusions can be made in light of the current study:

(1) The results of this study show that the application of the M5P technique for the prediction of the CS and STS of concrete made with SF yielded high predictive and generalization capabilities. A comparison of different techniques showed that M5P had superior predictive performance compared with linear regression analysis and gene expression programming for both the CS and STS databases;
(2) In the case of prediction of the CS using M5P, the values of *R*^2^ for both the training and testing sets were 0.82, while for the STS, the value of *R*^2^ was 0.88 for the training set and 0.86 for the testing set. For predicting both the CS and STS of concrete with SF, the accuracy of the prediction techniques for both training and testing sets was as follows: M5P > GEP > linear regression analysis;
(3) PA captured a linear correlation between the SF content and both the CS and STS. Both CS and STS increase by increasing the content of SF. For both the CS and STS of concrete with SF, it was observed that both parameters decreased by increasing the w/b ratio. In the case of CS, high early strength gain was observed owing to rapid SF pozzolanic reactions. While at later ages, the strength gain was not significant. For the STS, the strength gain was almost linear in the first 7 days and then increased nonlinearly with age, mostly up to 28 days.

## 7. Future Research

(1) In this study, only individual machine learning (ML) techniques were used for predicting the mechanical properties of concrete with silica fume. It would be beneficial to predict these properties of concrete with silica fume by using the ensemble machine learning technique and comparing it with individual techniques;
(2) In this study, parametric analysis (PA) was conducted and variation in mechanical properties was checked with only silica fume content, w/b ratio, and the age of specimens. In the future, it will be useful to conduct PA using a more accurate ML technique and to explore variations in mechanical properties with cement content, aggregate, and superplasticizer dosages as well. Moreover, sensitivity analysis needs to be investigated;
(3) We predicted only the compressive and splitting tensile strengths of concrete with silica by using ML techniques. Other properties, such as rheology, elastic modulus, flexural strength, and durability characteristics of concrete with silica fume, need to be predicted.

## Figures and Tables

**Table 1:** Descriptive statistics of the database used in the training dataset.

Compressive Strength Database								
Statistical Indicator	C (kg/m^3^)	SF (kg/m^3^)	W/B	FA (kg/m^3^)	CA (kg/m^3^)	SP (kg/m^3^)	Days	Strength (MPa)
Minimum	188	0	0.14	468.98	0	0	1	3.57
Maximum	1000	250	0.83	2750	1248	80	400	136.8
Mean	422.55	39.35	0.42	806.32	969.9	8.7	53.4	53.72
Standard error	4.44	1.37	0.005	10.02	11.51	0.46	3.11	0.83
Standard deviation	125.26	38.72	0.14	282.7	324.9	13.15	87.92	23.53
Kurtosis	2.54	7.27	0.6	21.15	3.15	13.75	6.53	−0.178
Skewness	0.99	2.13	0.92	3.85	−1.97	3.51	2.65	0.325
**Splitting Tensile Strength Database**								
Minimum	197	0	0.14	535	0	0	1	0.51
Maximum	800	250	0.83	1315	1248	80	91	10
Mean	458.02	54.36	0.38	816.58	892.56	13.19	32.11	4.23
Standard error	11.54	4.94	0.01	16.76	29.36	1.83	2.24	0.15
Standard deviation	144.07	61.65	0.14	209.34	366.71	22.83	27.95	1.87
Kurtosis	−0.07	2.46	1.31	−0.15	1.37	3.64	0.15	0.46
Skewness	0.73	1.76	0.67	0.85	−1.54	2.29	1.12	0.68

**Table 2:** Parameters of the developed GEP models.

Parameters	GEP Model for CS	GEP Model for STS
Head size	10	10
Chromosome	30	50
Genes	3	5
Linking function	Addition	Addition
Number of generations	50,000	50,000

**Table 3:** Coefficients of the linear models developed by the M5P for CS based on Equation (3).

Linear Model	a	b	c	d	e	f	g	h
**LM1**	78.471		0.0055	−222.5715	0.0343	0.0077	0.021	2.6966
**LM2**	61.8093		0.1844	−92.516	0.0277	0.0303	0.021	0.0331
**LM3**	87.4672		−0.0032	−161.529	0.0268	0.0134	0.021	0.0413
**LM4**	165.2335		−0.0091	−380.5541	0.0226	0.0134	0.021	0.0423
**LM5**	104.29		0.0955	−172.6348	0.0266	0.0134	0.021	0.0422
**LM6**	25.74	−0.003		−12.1069	0.0001	0.0005	0.7868	1.7661
**LM7**	28.5248	−0.003		−12.1069	0.0001	0.0005	0.7868	1.4339
**LM8**	31.443	−0.003		−12.1069	0.0001	0.0005	1.2469	1.5649
**LM9**	38.8668	−0.0022		−12.1069	0.0001	0.0005	0.4578	0.3137
**LM10**	45.222	−0.0022		−12.1069	0.0001	0.0005	0.5065	0.3137
**LM11**	22.314	−0.0017		−18.5849	0.0001	0.0005	0.2503	2.1477
**LM12**	46.1883		−0.0667	−15.2811	0.0163	0.0005	0.1293	0.0216
**LM13**	57.5337		−0.0667	−15.2811	0.0063	0.0005	0.1293	0.0216
**LM14**	59.0744		−0.0955	−15.2811	−0.0053	0.0005	0.1293	0.0216
**LM15**	54.7424		−0.1202	−15.2811	−0.0053	0.0005	0.1293	0.0216
**LM16**	67.5622		−0.0577	−15.2811	−0.0054	0.0005	0.1293	0.0296
**LM17**	83.8422		−0.0238	−62.7051	−0.0018	0.0005	0.1293	0.0224
**LM18**	75.9732			−13.91	−0.0043	0.0005	0.1795	0.0144
**LM19**	61.0595			−13.91	−0.0006	0.0005	0.1795	0.0144
**LM20**	66.9734			−13.91	−0.0014	0.0005	0.1795	0.0144

**Table 4:** Comparison of the different models developed for the CS database.

Models for CS	Training Set					Testing Set				
	*R*	*MAE*	*RSE*	*RMSE*	*DR* (%)	*R*	*MAE*	*RSE*	*RMSE*	*DR* (%)
Linear regression analysis	0.74	13.6	0.45	16.5	47	0.73	16	0.47	19.6	50
GEP	0.86	10.3	0.26	13.35	78	0.86	9.9	0.26	13.6	70
M5P	0.91	8.37	0.19	10.46	78.6	0.9	9	0.18	12.4	75

**Table 5:** Coefficients of the linear models developed by M5P for STS based on Equation (4).

Linear Model	a	b	c	d	e	f	g	h
**LM1**	15.27	−0.012	0.003	−17.43			0.0109	0.0293
**LM2**	−2.75	0.0113	0.0112	−5.02			0.0176	0.017
**LM3**	−3.877	0.0114	0.0056	2.1	0.0011		−0.0077	0.0173
**LM4**	11.53	−0.0034	0.0091	−15.12	−0.0006		−0.083	0.0173

**Table 6:** Comparison of the different models developed for the STS database.

Models for STS	Training Set					Testing Set				
	*R*	*MAE*	*RSE*	*RMSE*	*DR* (%)	*R*	*MAE*	*RSE*	*RMSE*	*DR* (%)
Linear regression analysis	0.81	1.1	0.37	1.23	46	0.83	0.84	0.32	1.1	68
GEP	0.88	0.71	0.22	0.9	75	0.84	0.83	0.3	1.09	66
M5P	0.94	0.51	0.14	0.64	75.3	0.93	0.7	0.17	0.85	75

**Table 7:** Porosity of concrete made with different contents of SF as partial cement replacement.

SF Content (%)	MIP Measured Total Porosity (%)			Reference
	7 Days	28 Days	90 Days	
**0**	8.4	7.9	7	[40]
5	7.2	6.3	5.8	
10	6.1	5.7	5.1	
0	7.8	6.5		[67]
10	6.7	6		

## Data Availability

The database used in this study can be found in Supplementary Materials.

## Supplementary Materials

The following supporting information can be downloaded at: https://www.mdpi.com/article/10.3390/ma15155436/s1, Table S1: Experimental database of compressive strength of concrete incorporating silica fume, Table S2: Experimental database of splitting tensile strength of concrete incorporating silica fume.
