# Supplementary material for: Predicting Compressive and Splitting Tensile Strengths of Silica Fume Concrete Using M5P Model Tree Algorithm
Source: Materials (Basel). 2022 Aug 7;15(15):5436. doi: 10.3390/ma15155436 (PMC9369534; doi:10.3390/ma15155436)
Supplement: Supplementary file 1 [file materials-15-05436-s001.zip › materials-1808414-supplementary.pdf]

Predicting Compressive and Splitting Tensile Strengths of Silica Fume Concrete Using M5P Model Tree Algorithm

Supplementary Information

Table S1. Experimental database of compressive strength of concrete incorporating silica fume.

S. No.	Cement (kg/m³)	Silica fume (kg/m³)	w/b	Fine aggregate (kg/m³)	Coarse aggregate (kg/m³)	Superplasticizer (kg/m³)	Days	Compressive strength (MPa)
1	475	23.75	0.3	719	1086	8	3	52.47
2	380	19	0.4	688	1157	5.5	3	35.8
3	389.5	19.47	0.5	605	1132	0	3	26.3
4	475	23.75	0.3	719	1086	8	7	75.5
5	380	19	0.4	688	1157	5.5	7	53
6	389.5	19.47	0.5	605	1132	0	7	39.2
7	475	23.75	0.3	719	1086	8	28	87.8
8	380	19	0.4	688	1157	5.5	28	69.4
9	389.5	19.47	0.5	605	1132	0	28	57.3
10	475	23.75	0.3	719	1086	8	56	93.1
11	380	19	0.4	688	1157	5.5	56	72.1
12	389.5	19.47	0.5	605	1132	0	56	59.6
13	475	23.75	0.3	719	1086	8	90	93.6
14	380	19	0.4	688	1157	5.5	90	73.7
15	389.5	19.47	0.5	605	1132	0	90	67.3
16	475	23.75	0.3	719	1086	8	180	99.3
17	380	19	0.4	688	1157	5.5	180	74.5

18	389.5	19.47	0.5	605	1132	0	180	66.3
19	600	0	0.3	1084	595	7.14	3	76
20	570	30	0.3	1072	595	7.98	3	73.7
21	540	60	0.3	1059	595	8.58	3	78.3
22	514	0	0.35	1131	621	7.71	3	65.5
23	489	26	0.35	1120	621	8.22	3	63.4
24	463	51	0.35	1110	621	9	3	70.8
25	450	0	0.4	1166	640	8.1	3	56.8
26	428	23	0.4	1157	640	8.55	3	55.6
27	405	45	0.4	1147	640	9.45	3	59.8
28	600	0	0.3	1084	595	7.14	7	71.37
29	570	30	0.3	1072	595	7.98	7	73.44
30	540	60	0.3	1059	595	8.58	7	76.05
31	514	0	0.35	1131	621	7.71	7	67.68
32	489	26	0.35	1120	621	8.22	7	69.84
33	463	51	0.35	1110	621	9	7	73.08
34	450	0	0.4	1166	640	8.1	7	59.04
35	428	23	0.4	1157	640	8.55	7	59.22
36	405	45	0.4	1147	640	9.45	7	62.73
37	600	0	0.3	1084	595	7.14	28	75.6
38	570	30	0.3	1072	595	7.98	28	85.77
39	540	60	0.3	1059	595	8.58	28	90.45
40	514	0	0.35	1131	621	7.71	28	74.7

41	489	26	0.3<br>5	1120	621	8.22	28	76.77
42	463	51	0.3<br>5	1110	621	9	28	84.24
43	450	0	0.4	1166	640	8.1	28	65.16
44	428	23	0.4	1157	640	8.55	28	67.77
45	405	45	0.4	1147	640	9.45	28	71.1
46	600	0	0.3	1084	595	7.14	90	79.47
47	570	30	0.3	1072	595	7.98	90	89.1
48	540	60	0.3	1059	595	8.58	90	95.94
49	514	0	0.3<br>5	1131	621	7.71	90	76.86
50	489	26	0.3<br>5	1120	621	8.22	90	81.81
51	463	51	0.3<br>5	1110	621	9	90	90.36
52	450	0	0.4	1166	640	8.1	90	72.36
53	428	23	0.4	1157	640	8.55	90	74.16
54	405	45	0.4	1147	640	9.45	90	77.49
55	385	0	0.4<br>6	911	214	0.96	3	19.7472
56	513	27	0.3	859	825	3.24	3	42.144
57	496.8	43.2	0.3	856	822	3.24	3	41.0784
58	480.6	59.4	0.3	853	820	3.24	3	41.7408
59	385	0	0.4<br>6	911	214	0.96	7	26.3616
60	513	27	0.3	859	825	3.24	7	51.0912
61	496.8	43.2	0.3	856	822	3.24	7	48.624
62	480.6	59.4	0.3	853	820	3.24	7	50.9856
63	450	0	0.2<br>8	675	1125	14	3	59.76

64	427.5	22.5	0.2 8	675	1125	14	3	66.69
65	405	45	0.2 8	675	1125	14	3	65.52
66	382.5	67.5	0.2 8	675	1125	14	3	65.16
67	450	0	0.2 8	675	1125	14	7	67.32
68	427.5	22.5	0.2 8	675	1125	14	7	77.31
69	405	45	0.2 8	675	1125	14	7	77.76
70	382.5	67.5	0.2 8	675	1125	14	7	78.3
71	450	0	0.2 8	675	1125	14	28	78.03
72	427.5	22.5	0.2 8	675	1125	14	28	95.13
73	405	45	0.2 8	675	1125	14	28	102.51
74	382.5	67.5	0.2 8	675	1125	14	28	105.75
75	450	0	0.2 8	675	1125	14	90	86.04
76	427.5	22.5	0.2 8	675	1125	14	90	103.59
77	405	45	0.2 8	675	1125	14	90	111.42
78	382.5	67.5	0.2 8	675	1125	14	90	114.21

79	450	0	0.28	675	1125	14	180	92.16
80	427.5	22.5	0.28	675	1125	14	180	109.53
81	405	45	0.28	675	1125	14	180	113.49
82	382.5	67.5	0.28	675	1125	14	180	115.47
83	450	0	0.28	675	1125	14	365	95.13
84	427.5	22.5	0.28	675	1125	14	365	111.69
85	405	45	0.28	675	1125	14	365	114.75
86	382.5	67.5	0.28	675	1125	14	365	116.19
87	427.5	22.5	0.36	990	735	8	28	58.04
88	405	45	0.38	990	735	8	28	62.81
89	382.5	67.5	0.4	990	735	8	28	67.95
90	360	90	0.4	990	735	8	28	66.35
91	390	0	0.47	569	1164	0	28	32.9
92	370.5	19.5	0.47	569	1164	0	28	34.8
93	351	39	0.47	569	1164	0	28	38.7
94	331.5	58.5	0.47	569	1164	0	28	36.6

95	550	0	0.32	645.14	1140.36	7.4	3	37.44
96	550	27.5	0.3	627.79	1109.71	7.66	3	37.92
97	550	55	0.3	610.45	1079.05	7.93	3	36.192
98	550	82.5	0.3	593.11	1048.4	8.6	3	34.56
99	600	0	0.3	613.6	1084.6	7.2	3	33.6
100	600	30	0.3	594.69	1051.1	7.46	3	31.296
101	600	60	0.3	585.55	1017.7	7.8	3	33.504
102	600	90	0.3	556.85	984.3	8.13	3	31.968
103	550	0	0.32	645.14	1140.36	7.4	7	44.16
104	550	27.5	0.3	627.79	1109.71	7.66	7	48.48
105	550	55	0.3	610.45	1079.05	7.93	7	48.96
106	550	82.5	0.3	593.11	1048.4	8.6	7	49.152
107	600	0	0.3	613.6	1084.6	7.2	7	46.464
108	600	30	0.3	594.69	1051.1	7.46	7	43.2
109	600	60	0.3	585.55	1017.7	7.8	7	43.2
110	600	90	0.3	556.85	984.3	8.13	7	42.624
111	550	0	0.32	645.14	1140.36	7.4	28	53.73
112	550	27.5	0.3	627.79	1109.71	7.66	28	61.29
113	550	55	0.3	610.45	1079.05	7.93	28	59.85
114	550	82.5	0.3	593.11	1048.4	8.6	28	65.07
115	600	0	0.3	613.6	1084.6	7.2	28	52.02
116	600	30	0.3	594.69	1051.1	7.46	28	55.26
117	600	60	0.3	585.55	1017.7	7.8	28	57.06
118	600	90	0.3	556.85	984.3	8.13	28	54
119	400	30	0.41	836	836	14.3	7	28.8
120	400	40	0.41	825	825	14.4	7	29.2

121	400	60	0.41	804	804	14.6	7	31
122	500	50	0.41	725	725	14.7	7	37
123	400	30	0.41	836	836	14.3	28	39.5
124	400	40	0.41	825	825	14.4	28	42.25
125	400	60	0.41	804	804	14.6	28	45
126	500	50	0.41	725	725	14.7	28	48
127	400	30	0.41	836	836	14.3	56	44.25
128	400	40	0.41	825	825	14.4	56	47
129	400	60	0.41	804	804	14.6	56	48
130	500	50	0.41	725	725	14.7	56	49.5
131	235	0	0.79	700	1200	0.705	7	17.76
132	223.25	11.75	0.79	700	1200	1.18	7	18.24
133	211.5	23.5	0.79	700	1200	1.7	7	18.72
134	199.75	35.25	0.79	700	1200	2.26	7	19.2
135	188	47	0.79	700	1200	2.7	7	19.2

136	285	0	0.65	730	1200	0.68	7	25.44
137	270.75	14.25	0.65	730	1200	1.4	7	26.88
138	256.5	28.5	0.65	730	1200	1.97	7	26.88
139	242.25	42.75	0.65	730	1200	2.78	7	27.84
140	228	57	0.65	730	1200	3.28	7	28.32
141	310	0	0.6	710	1200	0.4	7	29.28
142	294.5	15.5	0.6	710	1200	0.88	7	31.2
143	279	31	0.6	710	1200	1.7	7	31.68
144	263.5	46.5	0.6	710	1200	2.74	7	32.64
145	248	62	0.6	710	1200	3.22	7	33.12
146	355	0	0.52	670	1200	0.4	7	36.96
147	337.25	17.75	0.52	670	1200	1.85	7	39.36
148	319.5	35.5	0.52	670	1200	3.35	7	41.76
149	301.75	53.25	0.52	670	1200	4.16	7	42.24
150	284	71	0.52	670	1200	4.14	7	43.68
151	410	0	0.45	625	1200	0.86	7	45.6
152	389.5	20.5	0.45	625	1200	3.27	7	48.48
153	369	41	0.45	625	1200	4.35	7	53.28

154	348.5	61.5	0.45	625	1200	4.67	7	51.3
155	328	82	0.45	625	1200	4.95	7	52.2
156	235	0	0.79	700	1200	0.705	28	27.5
157	223.25	11.75	0.79	700	1200	1.18	28	30
158	211.5	23.5	0.79	700	1200	1.7	28	32
159	199.75	35.25	0.79	700	1200	2.26	28	33
160	188	47	0.79	700	1200	2.7	28	33.5
161	285	0	0.65	730	1200	0.68	28	37
162	270.75	14.25	0.65	730	1200	1.4	28	41.5
163	256.5	28.5	0.65	730	1200	1.97	28	45
164	242.25	42.75	0.65	730	1200	2.78	28	47
165	228	57	0.65	730	1200	3.28	28	48
166	310	0	0.6	710	1200	0.4	28	41
167	294.5	15.5	0.6	710	1200	0.88	28	46.5
168	279	31	0.6	710	1200	1.7	28	50.5
169	263.5	46.5	0.6	710	1200	2.74	28	52.5
170	248	62	0.6	710	1200	3.22	28	54
171	355	0	0.52	670	1200	0.4	28	50

172	337.25	17.75	0.52	670	1200	1.85	28	57
173	319.5	35.5	0.52	670	1200	3.35	28	62
174	301.75	53.25	0.52	670	1200	4.16	28	64
175	284	71	0.52	670	1200	4.14	28	66
176	410	0	0.45	625	1200	0.86	28	59
177	389.5	20.5	0.45	625	1200	3.27	28	68
178	369	41	0.45	625	1200	4.35	28	74.5
179	348.5	61.5	0.45	625	1200	4.67	28	77
180	328	82	0.45	625	1200	4.95	28	79
181	235	0	0.79	700	1200	0.705	60	31
182	223.25	11.75	0.79	700	1200	1.18	60	33.5
183	211.5	23.5	0.79	700	1200	1.7	60	35.5
184	199.75	35.25	0.79	700	1200	2.26	60	36.5
185	188	47	0.79	700	1200	2.7	60	37.5
186	285	0	0.65	730	1200	0.68	60	41

187	270.75	14.25	0.65	730	1200	1.4	60	45
188	256.5	28.5	0.65	730	1200	1.97	60	48.5
189	242.25	42.75	0.65	730	1200	2.78	60	50
190	228	57	0.65	730	1200	3.28	60	50.5
191	310	0	0.6	710	1200	0.4	60	45
192	294.5	15.5	0.6	710	1200	0.88	60	50.5
193	279	31	0.6	710	1200	1.7	60	54.5
194	263.5	46.5	0.6	710	1200	2.74	60	56
195	248	62	0.6	710	1200	3.22	60	57
196	355	0	0.52	670	1200	0.4	60	54
197	337.25	17.75	0.52	670	1200	1.85	60	61
198	319.5	35.5	0.52	670	1200	3.35	60	66
199	301.75	53.25	0.52	670	1200	4.16	60	67.5
200	284	71	0.52	670	1200	4.14	60	69
201	410	0	0.45	625	1200	0.86	60	63.5
202	389.5	20.5	0.45	625	1200	3.27	60	72
203	369	41	0.45	625	1200	4.35	60	78.5
204	348.5	61.5	0.45	625	1200	4.67	60	80.5

205	328	82	0.45	625	1200	4.95	60	82
206	235	0	0.79	700	1200	0.705	180	34
207	223.25	11.75	0.79	700	1200	1.18	180	36.5
208	211.5	23.5	0.79	700	1200	1.7	180	39
209	199.75	35.25	0.79	700	1200	2.26	180	40
210	188	47	0.79	700	1200	2.7	180	40
211	285	0	0.65	730	1200	0.68	180	44.5
212	270.75	14.25	0.65	730	1200	1.4	180	49
213	256.5	28.5	0.65	730	1200	1.97	180	52.5
214	242.25	42.75	0.65	730	1200	2.78	180	53
215	228	57	0.65	730	1200	3.28	180	53.5
216	310	0	0.6	710	1200	0.4	180	49
217	294.5	15.5	0.6	710	1200	0.88	180	54.5
218	279	31	0.6	710	1200	1.7	180	58
219	263.5	46.5	0.6	710	1200	2.74	180	59

220	248	62	0.6	710	1200	3.22	180	59.5
221	355	0	0.52	670	1200	0.4	180	58.5
222	337.25	17.75	0.52	670	1200	1.85	180	65
223	319.5	35.5	0.52	670	1200	3.35	180	71
224	301.75	53.25	0.52	670	1200	4.16	180	72.5
225	284	71	0.52	670	1200	4.14	180	73.5
226	410	0	0.45	625	1200	0.86	180	68
227	389.5	20.5	0.45	625	1200	3.27	180	75.5
228	369	41	0.45	625	1200	4.35	180	83
229	348.5	61.5	0.45	625	1200	4.67	180	85.5
230	328	82	0.45	625	1200	4.95	180	85.5
231	235	0	0.79	700	1200	0.705	365	35
232	223.25	11.75	0.79	700	1200	1.18	365	37.5
233	211.5	23.5	0.79	700	1200	1.7	365	40
234	199.75	35.25	0.79	700	1200	2.26	365	41

235	188	47	0.79	700	1200	2.7	365	41
236	285	0	0.65	730	1200	0.68	365	46.5
237	270.75	14.25	0.65	730	1200	1.4	365	50.5
238	256.5	28.5	0.65	730	1200	1.97	365	54
239	242.25	42.75	0.65	730	1200	2.78	365	54.5
240	228	57	0.65	730	1200	3.28	365	55
241	310	0	0.6	710	1200	0.4	365	50.5
242	294.5	15.5	0.6	710	1200	0.88	365	56
243	279	31	0.6	710	1200	1.7	365	59.5
244	263.5	46.5	0.6	710	1200	2.74	365	60.5
245	248	62	0.6	710	1200	3.22	365	61
246	355	0	0.52	670	1200	0.4	365	61
247	337.25	17.75	0.52	670	1200	1.85	365	67
248	319.5	35.5	0.52	670	1200	3.35	365	72.5
249	301.75	53.25	0.52	670	1200	4.16	365	73.5

250	284	71	0.52	670	1200	4.14	365	74.5
251	410	0	0.45	625	1200	0.86	365	71
252	389.5	20.5	0.45	625	1200	3.27	365	78
253	369	41	0.45	625	1200	4.35	365	84.5
254	348.5	61.5	0.45	625	1200	4.67	365	86
255	328	82	0.45	625	1200	4.95	365	87
256	500	0	0.35	647	1203	8.17	7	41.4
257	470	30	0.35	647	1203	9.78	7	45.45
258	450	50	0.35	647	1203	11.71	7	46.8
259	425	75	0.35	647	1203	13.34	7	47.7
260	500	0	0.35	647	1203	8.17	14	46.8
261	470	30	0.35	647	1203	9.78	14	52.2
262	450	50	0.35	647	1203	11.71	14	54.9
263	425	75	0.35	647	1203	13.34	14	56.7
264	500	0	0.35	647	1203	8.17	28	52.2

265	470	30	0.35	647	1203	9.78	28	58.5
266	450	50	0.35	647	1203	11.71	28	60.75
267	425	75	0.35	647	1203	13.34	28	63
268	500	0	0.35	647	1203	8.17	90	57.6
269	470	30	0.35	647	1203	9.78	90	63.9
270	450	50	0.35	647	1203	11.71	90	66.6
271	425	75	0.35	647	1203	13.34	90	68.4
272	500	0	0.35	647	1203	8.17	365	65.7
273	470	30	0.35	647	1203	9.78	365	65.7
274	450	50	0.35	647	1203	11.71	365	66.6
275	425	75	0.35	647	1203	13.34	365	67.5
276	385	0	0.46	920	884	2.31	7	31.632
277	354.2	30.8	0.46	915	878	2.7	7	34.35725
278	450	0	0.36	912	877	4.95	7	43.84973
279	414	36	0.36	906	870	5.4	7	47.1767

280	385	0	0.46	920	884	2.31	28	38.06208
281	354.2	30.8	0.46	915	878	2.7	28	45.96941
282	450	0	0.36	912	877	4.95	28	51.22253
283	414	36	0.36	906	870	5.4	28	54.72576
284	385	0	0.46	920	884	2.31	90	42.99264
285	354.2	30.8	0.46	915	878	2.7	90	49.62816
286	450	0	0.36	912	877	4.95	90	52.71264
287	414	36	0.36	906	870	5.4	90	60.03072
288	426	22	0.34	630	1169	2.2	3	25.74
289	449	50	0.28	622	1155	5	3	37.98
290	468	83	0.24	612	1136	8.3	3	56.7
291	478	120	0.2	603	1119	12	3	76.41
292	426	22	0.34	630	1169	2.2	7	45.09
293	449	50	0.28	622	1155	5	7	60.48
294	468	83	0.24	612	1136	8.3	7	76.32
295	478	120	0.2	603	1119	12	7	92.25

296	426	22	0.34	630	1169	2.2	28	54
297	449	50	0.28	622	1155	5	28	72
298	468	83	0.24	612	1136	8.3	28	90
299	478	120	0.2	603	1119	12	28	108
300	426	22	0.34	630	1169	2.2	1	15.12
301	449	50	0.28	622	1155	5	1	21.69
302	468	83	0.24	612	1136	8.3	1	30.96
303	478	120	0.2	603	1119	12	1	40.59
304	500	0	0.27	720	1050	43	3	61.2
305	475	25	0.27	725	1050	43	3	56.7
306	450	50	0.27	715	1050	43	3	54.9
307	425	75	0.27	715	1050	43	3	53.55
308	500	0	0.3	695	1050	19	3	57.15
309	475	25	0.3	685	1050	19	3	55.8
310	450	50	0.3	680	1050	19	3	55.35
311	425	75	0.3	680	1050	19	3	51.75
312	500	0	0.33	700	1050	12	3	52.2
313	475	25	0.33	690	1050	12	3	49.5

314	450	50	0.33	685	1050	12	3	47.7
315	425	75	0.33	680	1050	12	3	42.75
316	500	0	0.27	720	1050	43	7	65.25
317	475	25	0.27	725	1050	43	7	67.95
318	450	50	0.27	715	1050	43	7	71.1
319	425	75	0.27	715	1050	43	7	68.85
320	500	0	0.3	695	1050	19	7	64.8
321	475	25	0.3	685	1050	19	7	72.9
322	450	50	0.3	680	1050	19	7	70.65
323	425	75	0.3	680	1050	19	7	67.05
324	500	0	0.33	700	1050	12	7	56.25
325	475	25	0.33	690	1050	12	7	62.55
326	450	50	0.33	685	1050	12	7	63.45
327	425	75	0.33	680	1050	12	7	63.45
328	500	0	0.27	720	1050	43	28	75.6
329	475	25	0.27	725	1050	43	28	79.65
330	450	50	0.27	715	1050	43	28	85.95

331	425	75	0.27	715	1050	43	28	90.9
332	500	0	0.3	695	1050	19	28	75.15
333	475	25	0.3	685	1050	19	28	81.9
334	450	50	0.3	680	1050	19	28	85.5
335	425	75	0.3	680	1050	19	28	88.65
336	500	0	0.33	700	1050	12	28	67.5
337	475	25	0.33	690	1050	12	28	62.55
338	450	50	0.33	685	1050	12	28	63.45
339	425	75	0.33	680	1050	12	28	63.45
340	500	0	0.27	720	1050	43	56	77.85
341	475	25	0.27	725	1050	43	56	83.7
342	450	50	0.27	715	1050	43	56	90
343	425	75	0.27	715	1050	43	56	93.15
344	500	0	0.3	695	1050	19	56	76.05
345	475	25	0.3	685	1050	19	56	85.95
346	450	50	0.3	680	1050	19	56	87.3
347	425	75	0.3	680	1050	19	56	91.35
348	500	0	0.33	700	1050	12	56	70.2
349	475	25	0.33	690	1050	12	56	76.5

350	450	50	0.33	685	1050	12	56	81.45
351	425	75	0.33	680	1050	12	56	83.7
352	500	0	0.27	720	1050	43	90	78.75
353	475	25	0.27	725	1050	43	90	86.85
354	450	50	0.27	715	1050	43	90	93.6
355	425	75	0.27	715	1050	43	90	95.4
356	500	0	0.3	695	1050	19	90	76.95
357	475	25	0.3	685	1050	19	90	85.95
358	450	50	0.3	680	1050	19	90	89.1
359	425	75	0.3	680	1050	19	90	93.6
360	500	0	0.33	700	1050	12	90	71.1
361	475	25	0.33	690	1050	12	90	81
362	450	50	0.33	685	1050	12	90	82.8
363	425	75	0.33	680	1050	12	90	85.95
364	500	0	0.27	720	1050	43	180	81
365	475	25	0.27	725	1050	43	180	87.75
366	450	50	0.27	715	1050	43	180	96.3

367	425	75	0.27	715	1050	43	180	98.1
368	500	0	0.3	695	1050	19	180	78.75
369	475	25	0.3	685	1050	19	180	87.75
370	450	50	0.3	680	1050	19	180	92.7
371	425	75	0.3	680	1050	19	180	95.85
372	500	0	0.33	700	1050	12	180	73.35
373	475	25	0.33	690	1050	12	180	81
374	450	50	0.33	685	1050	12	180	84.15
375	425	75	0.33	680	1050	12	180	90.45
376	500	0	0.27	720	1050	43	1	35.1
377	475	25	0.27	725	1050	43	1	31.5
378	450	50	0.27	715	1050	43	1	22.5
379	425	75	0.27	715	1050	43	1	22.05
380	500	0	0.3	695	1050	19	1	43.2
381	475	25	0.3	685	1050	19	1	41.4
382	450	50	0.3	680	1050	19	1	37.8
383	425	75	0.3	680	1050	19	1	34.2

384	500	0	0.33	700	1050	12	1	36.9
385	475	25	0.33	690	1050	12	1	31.5
386	450	50	0.33	685	1050	12	1	28.8
387	425	75	0.33	680	1050	12	1	27.9
388	385	0	0.46	920	884	2.31	7	32.95
389	354.2	30.8	0.46	915	879	2.7	7	37.28
390	450	0	0.36	912	877	4.95	7	47.58
391	414	36	0.36	906	871	5.4	7	51.19
392	385	0	0.46	920	884	2.31	28	41.3
393	354.2	30.8	0.46	915	879	2.7	28	49.88
394	450	0	0.36	912	877	4.95	28	55.58
395	414	36	0.36	906	871	5.4	28	63.34
396	385	0	0.46	920	884	2.31	90	46.65
397	354.2	30.8	0.46	915	879	2.7	90	57.44
398	450	0	0.36	912	877	4.95	90	61.01

399	414	36	0.36	906	871	5.4	90	69.48
400	385	0	0.46	920	884	2.31	365	52.57
401	354.2	30.8	0.46	915	879	2.7	365	60.13
402	450	0	0.36	912	877	4.95	365	68.33
403	414	36	0.36	906	871	5.4	365	76.24
404	315	215	0.45	960	813	6.2	7	27.33
405	357	196	0.4	960	813	6.3	7	32.29
406	411	135	0.35	960	813	7.29	7	44.6
407	315	215	0.45	960	813	6.2	28	45.11
408	357	196	0.4	960	813	6.3	28	57.11
409	411	135	0.35	960	813	7.29	28	61.12
410	500	0	0.3	725	1087	0.5	3	61.65
411	475	25	0.3	716	1087	0.6	3	60.3
412	450	50	0.3	707	1087	0.8	3	56.88
413	410	0	0.5	662	1081	0	3	25.74
414	390	20.5	0.5	655	1081	0	3	24.66
415	369	41	0.5	648	1081	0	3	23.22
416	500	0	0.3	725	1087	0.5	7	72.99
417	475	25	0.3	716	1087	0.6	7	71.37
418	450	50	0.3	707	1087	0.8	7	69.21
419	410	0	0.5	662	1081	0	7	37.08
420	390	20.5	0.5	655	1081	0	7	42.3

421	369	41	0.5	648	1081	0	7	42.66
422	500	0	0.3	725	1087	0.5	28	86.85
423	475	25	0.3	716	1087	0.6	28	95.85
424	450	50	0.3	707	1087	0.8	28	97.11
425	410	0	0.5	662	1081	0	28	46.89
426	390	20.5	0.5	655	1081	0	28	48.87
427	369	41	0.5	648	1081	0	28	52.56
428	500	0	0.3	725	1087	0.5	90	92.25
429	475	25	0.3	716	1087	0.6	90	99.18
430	450	50	0.3	707	1087	0.8	90	104.04
431	410	0	0.5	662	1081	0	90	54.36
432	390	20.5	0.5	655	1081	0	90	60.75
433	369	41	0.5	648	1081	0	90	62.19
434	450	0	0.4	688	1062	13.5	90	75.8
435	428	22.5	0.4	685	1058	13.5	90	81
436	405	45	0.4	682	1054	13.5	90	82.7
437	383	67.5	0.4	680	1049	13.5	90	84
438	360	90	0.4	677	1045	13.5	90	85.7
439	350	0	0.6	697	1076	5.3	90	53.8
440	333	17.5	0.6	695	1073	5.3	90	56.8
441	315	35	0.6	693	1070	5.3	90	57.7
442	298	52.5	0.6	691	1066	5.3	90	60.3
443	280	70	0.6	688	1063	5.3	90	59.7
444	1000	0	0.62	2750	0	0	7	21.58
445	950	50	0.62	2750	0	0	7	21.82
446	925	75	0.63	2750	0	0	7	22.54
447	900	100	0.64	2750	0	0	7	24.49

448	522.96	0	0.4	497.265	1196.07	0	7	30.01
449	496.81	26.148	0.4	486.57	1196.07	0	7	30.32
450	474.06	38.44	0.4	474.22	1196.07	0	7	32.01
451	461.25	51.25	0.4	468.98	1196.07	0	7	35.9
452	1000	0	0.62	2750	0	0	28	32.37
453	950	50	0.62	2750	0	0	28	32.85
454	925	75	0.63	2750	0	0	28	32.84
455	900	100	0.64	2750	0	0	28	33.09
456	522.96	0	0.4	497.265	1196.07	0	28	41.84
457	496.81	26.148	0.4	486.57	1196.07	0	28	42.32
458	474.06	38.44	0.4	474.22	1196.07	0	28	42.31
459	461.25	51.25	0.4	468.98	1196.07	0	28	43.31
460	494	0	0.32	647	1151	4.94	28	61.5
461	479.2	14.8	0.32	647	1151	4.94	28	62.6
462	464.4	29.6	0.32	647	1151	4.94	28	64.7
463	449.5	44.5	0.32	647	1151	4.94	28	66.6
464	434.7	59.3	0.32	647	1151	4.94	28	68.3
465	370	0	0.38	780	1180	4	3	26.32
466	351.5	18.5	0.38	780	1180	4	3	28.11

467	333	37	0.38	780	1180	4	3	30.57
468	314.5	55.5	0.38	780	1180	4	3	29.19
469	296	74	0.38	780	1180	4	3	28.02
470	277.5	92.5	0.38	780	1180	4	3	26.39
471	370	0	0.38	780	1180	4	7	30.55
472	351.5	18.5	0.38	780	1180	4	7	33.11
473	333	37	0.38	780	1180	4	7	38.26
474	314.5	55.5	0.38	780	1180	4	7	34.59
475	296	74	0.38	780	1180	4	7	31.4
476	277.5	92.5	0.38	780	1180	4	7	30.85
477	370	0	0.38	780	1180	4	14	36.07
478	351.5	18.5	0.38	780	1180	4	14	40.77
479	333	37	0.38	780	1180	4	14	44.72
480	314.5	55.5	0.38	780	1180	4	14	42.58
481	296	74	0.38	780	1180	4	14	36.25

482	277.5	92.5	0.38	780	1180	4	14	36.2
483	370	0	0.38	780	1180	4	28	40.55
484	351.5	18.5	0.38	780	1180	4	28	44.44
485	333	37	0.38	780	1180	4	28	48.75
486	314.5	55.5	0.38	780	1180	4	28	45.17
487	296	74	0.38	780	1180	4	28	41.53
488	277.5	92.5	0.38	780	1180	4	28	10.9
489	378.85	0	0.52	718.48	1224.97	0	7	18.96
490	359.95	18.9	0.52	718.48	1224.97	0	7	19.03
491	340.97	37.88	0.52	718.48	1224.97	0	7	19.77
492	322.05	56.8	0.52	718.48	1224.97	0	7	15.92
493	303.08	75.77	0.52	718.48	1224.97	0	7	14.66
494	378.85	0	0.52	718.48	1224.97	0	28	27.7
495	359.95	18.9	0.52	718.48	1224.97	0	28	28.14
496	340.97	37.88	0.52	718.48	1224.97	0	28	29.18

497	322.05	56.8	0.52	718.48	1224.97	0	28	23.25
498	303.08	75.77	0.52	718.48	1224.97	0	28	21.03
499	378.85	0	0.52	718.48	1224.97	0	56	39.84
500	359.95	18.9	0.52	718.48	1224.97	0	56	39.84
501	340.97	37.88	0.52	718.48	1224.97	0	56	42.21
502	322.05	56.8	0.52	718.48	1224.97	0	56	29.92
503	303.08	75.77	0.52	718.48	1224.97	0	56	24.29
504	378.85	0	0.52	718.48	1224.97	0	90	40.88
505	359.95	18.9	0.52	718.48	1224.97	0	90	41.03
506	340.97	37.88	0.52	718.48	1224.97	0	90	43.77
507	322.05	56.8	0.52	718.48	1224.97	0	90	32.44
508	303.08	75.77	0.52	718.48	1224.97	0	90	24.44
509	400	0	0.4	677.5	1204.4	8	28	50.7
510	380	20	0.4	674.11	1212.34	8	28	49.42
511	360	40	0.4	666.28	1212.34	8	28	63.05
512	340	60	0.4	658.46	1212.34	8	28	52.27
513	511	0	0.29	773	1044	5.11	7	53.6

514	486	25.55	0.29	773	1044	5.11	7	56.4
515	461	51.1	0.29	773	1044	5.11	7	58.88
516	436	76.65	0.29	773	1044	5.11	7	60
517	411	102.2	0.29	773	1044	5.11	7	58.88
518	386	127.75	0.29	773	1044	5.11	7	56.8
519	511	0	0.29	773	1044	5.11	28	59.55
520	486	25.55	0.29	773	1044	5.11	28	62.67
521	461	51.1	0.29	773	1044	5.11	28	65.33
522	436	76.65	0.29	773	1044	5.11	28	71.11
523	411	102.2	0.29	773	1044	5.11	28	67.33
524	386	127.75	0.29	773	1044	5.11	28	63.11
525	440	0	0.36	760	1050	8.8	28	42.59
526	418	22	0.36	760	1050	8.36	28	50.5
527	396	44	0.36	760	1050	7.92	28	53.72
528	650	0	0.4	1490	0	5.2	3	22.6
529	637	13	0.4	1490	0	5.525	3	23
530	624	26	0.4	1490	0	6.2	3	24.3

531	611	39	0.4	1490	0	6.8	3	25.7
532	650	0	0.5	1425	0	2.9	3	15.8
533	637	13	0.5	1425	0	3.25	3	15.9
534	624	26	0.5	1425	0	3.9	3	16.2
535	611	39	0.5	1425	0	3.9	3	17.3
536	650	0	0.4	1490	0	5.2	7	41.9
537	637	13	0.4	1490	0	5.525	7	43.3
538	624	26	0.4	1490	0	6.2	7	44.8
539	611	39	0.4	1490	0	6.8	7	46.2
540	650	0	0.5	1425	0	2.9	7	28.6
541	637	13	0.5	1425	0	3.25	7	29
542	624	26	0.5	1425	0	3.9	7	31
543	611	39	0.5	1425	0	3.9	7	32.9
544	650	0	0.4	1490	0	5.2	14	46.4
545	637	13	0.4	1490	0	5.525	14	47
546	624	26	0.4	1490	0	6.2	14	47.9
547	611	39	0.4	1490	0	6.8	14	50.1
548	650	0	0.5	1425	0	2.9	14	31.5
549	637	13	0.5	1425	0	3.25	14	32.4
550	624	26	0.5	1425	0	3.9	14	34.1
551	611	39	0.5	1425	0	3.9	14	36.2
552	650	0	0.4	1490	0	5.2	28	49.7
553	637	13	0.4	1490	0	5.525	28	49.6
554	624	26	0.4	1490	0	6.2	28	51
555	611	39	0.4	1490	0	6.8	28	52.4
556	650	0	0.5	1425	0	2.9	28	34.9
557	637	13	0.5	1425	0	3.25	28	35.3
558	624	26	0.5	1425	0	3.9	28	37.2
559	611	39	0.5	1425	0	3.9	28	38.4
560	520	0	0.3	860	886	5.2	7	64.08
561	468	52	0.3	851	877	5.2	7	67.68

562	520	0	0.3	860	886	5.2	28	74.34
563	468	52	0.3	851	877	5.2	28	79.92
564	520	0	0.3	860	886	5.2	91	79.47
565	468	52	0.3	851	877	5.2	91	88.2
566	500	0	0.35	900	600	17.5	7	20
567	350	150	0.35	900	600	10.5	7	34
568	300	200	0.35	900	600	10.35	7	32
569	250	250	0.35	900	600	10	7	30.15
570	700	0	0.18	1230	0	70	7	49.5
571	700	105	0.156	1230	0	70	7	79.2
572	700	210	0.14	1230	0	70	7	95.4
573	750	0	0.18	1230	0	75	7	58.95
574	750	112.5	0.156	1230	0	75	7	95.4
575	750	225	0.14	1230	0	75	7	115.65
576	800	0	0.18	1230	0	80	7	65.7
577	800	120	0.156	1230	0	80	7	108.9
578	800	240	0.14	1230	0	80	7	133.2

579	500	0	0.3<br>5	900	600	17.5	28	30
580	350	150	0.3<br>5	900	600	10.5	28	48.88
581	300	200	0.3<br>5	900	600	10.35	28	42.23
582	250	250	0.3<br>5	900	600	10	28	35.14
583	700	0	0.1<br>8	1230	0	70	28	60.3
584	700	105	0.1<br>56	1230	0	70	28	85.95
585	700	210	0.1<br>4	1230	0	70	28	102.6
586	750	0	0.1<br>8	1230	0	75	28	66.6
587	750	112.5	0.1<br>56	1230	0	75	28	100.35
588	750	225	0.1<br>4	1230	0	75	28	120.6
589	800	0	0.1<br>8	1230	0	80	28	68.4
590	800	120	0.1<br>56	1230	0	80	28	112.5
591	800	240	0.1<br>4	1230	0	80	28	136.8
592	600	0	0.3<br>3	900	750	12	7	63.99
593	570	30	0.3<br>3	900	750	12	7	63.324

594	540	60	0.32	900	750	12	7	61.029
595	600	0	0.33	900	750	12	28	68.76
596	570	30	0.33	900	750	12	28	72.378
597	540	60	0.32	900	750	12	28	71.262
598	600	0	0.33	900	750	12	90	73.638
599	570	30	0.33	900	750	12	90	80.784
600	540	60	0.32	900	750	12	90	77.472
601	450	0	0.4	670	1100	1.125	7	60.678
602	405	45	0.4	670	1100	4.5	7	67.986
603	450	0	0.4	670	1100	1.125	28	76.401
604	405	45	0.4	670	1100	4.5	28	84.924
605	450	0	0.4	670	1100	1.125	56	82.044
606	405	45	0.4	670	1100	4.5	56	88.11
607	450	0	0.4	670	1100	1.125	90	83.052
608	405	45	0.4	670	1100	4.5	90	90.36
609	495	24.75	0.3	535	1248	5.13	7	50.24
610	470	24.75	0.3	535	1248	5.13	7	56.22
611	495	24.75	0.3	535	1248	5.13	28	73.9
612	470	24.75	0.3	535	1248	5.13	28	78.96
613	495	24.75	0.3	535	1248	5.13	56	79.2
614	470	24.75	0.3	535	1248	5.13	56	85.3
615	610	50	0.22	631	1070	7.75	3	80.217

616	500	50	0.31	656	1070	3.25	3	62.937
617	286	32	0.55	843	1070	0	3	33.84
618	197	21	0.83	916	1070	0	3	13.383
619	610	50	0.22	631	1070	7.75	7	90.243
620	500	50	0.31	656	1070	3.25	7	76.14
621	286	32	0.55	843	1070	0	7	43.263
622	197	21	0.83	916	1070	0	7	18.243
623	610	50	0.22	631	1070	7.75	28	105.597
624	500	50	0.31	656	1070	3.25	28	89.703
625	286	32	0.55	843	1070	0	28	57.303
626	197	21	0.83	916	1070	0	28	28.647
627	610	50	0.22	631	1070	7.75	1	54.18
628	500	50	0.31	656	1070	3.25	1	41.697
629	286	32	0.55	843	1070	0	1	10.197
630	197	21	0.83	916	1070	0	1	3.573
631	450	0	0.4	630.75	1097.23	2.25	1	23.7

632	427.5	22.5	0.4	630.75	1097.23	4.5	1	31.39
633	405	45	0.4	630.75	1097.23	7.2	1	34.44
634	382.5	67.5	0.4	630.75	1097.23	9.45	1	40.84
635	360	90	0.4	630.75	1097.23	11.7	1	45.04
636	450	0	0.4	630.75	1097.23	2.25	7	30.67
637	427.5	22.5	0.4	630.75	1097.23	4.5	7	40.4
638	405	45	0.4	630.75	1097.23	7.2	7	40.26
639	382.5	67.5	0.4	630.75	1097.23	9.45	7	40.74
640	360	90	0.4	630.75	1097.23	11.7	7	38.1
641	450	0	0.4	630.75	1097.23	2.25	28	49.48
642	427.5	22.5	0.4	630.75	1097.23	4.5	28	56.03
643	405	45	0.4	630.75	1097.23	7.2	28	62.67
644	382.5	67.5	0.4	630.75	1097.23	9.45	28	62.37
645	360	90	0.4	630.75	1097.23	11.7	28	67.72
646	450	0	0.44	599	1148	0	28	39.88
647	432	18	0.44	599	1148	0	28	42.78
648	414	36	0.44	599	1148	0	28	45.95
649	396	54	0.44	599	1148	0	28	44.18
650	235	0	0.79	700	1200	0.705	1	7.5
651	223.25	11.75	0.79	700	1200	1.18	1	7
652	211.5	23.5	0.79	700	1200	1.7	1	6
653	199.75	35.25	0.79	700	1200	2.26	1	5

654	188	47	0.79	700	1200	2.7	1	4
655	285	0	0.65	730	1200	0.68	1	10.5
656	270.75	14.25	0.65	730	1200	1.4	1	10
657	256.5	28.5	0.65	730	1200	1.97	1	9.5
658	242.25	42.75	0.65	730	1200	2.78	1	9
659	228	57	0.65	730	1200	3.28	1	8.5
660	310	0	0.6	710	1200	0.4	1	12
661	294.5	15.5	0.6	710	1200	0.88	1	11.5
662	279	31	0.6	710	1200	1.7	1	11
663	263.5	46.5	0.6	710	1200	2.74	1	10.5
664	248	62	0.6	710	1200	3.22	1	10
665	355	0	0.52	670	1200	0.4	1	16
666	337.25	17.75	0.52	670	1200	1.85	1	15.5
667	319.5	35.5	0.52	670	1200	3.35	1	15
668	301.75	53.25	0.52	670	1200	4.16	1	14.5
669	284	71	0.52	670	1200	4.14	1	14
670	410	0	0.45	625	1200	0.86	1	20.5
671	389.5	20.5	0.45	625	1200	3.27	1	20.5

672	369	41	0.45	625	1200	4.35	1	19.5
673	348.5	61.5	0.45	625	1200	4.67	1	19
674	328	82	0.45	625	1200	4.95	1	18.5
675	235	0	0.79	700	1200	0.705	2	12
676	223.25	11.75	0.79	700	1200	1.18	2	11.5
677	211.5	23.5	0.79	700	1200	1.7	2	11
678	199.75	35.25	0.79	700	1200	2.26	2	10.5
679	188	47	0.79	700	1200	2.7	2	9.5
680	285	0	0.65	730	1200	0.68	2	17.5
681	270.75	14.25	0.65	730	1200	1.4	2	17
682	256.5	28.5	0.65	730	1200	1.97	2	16.5
683	242.25	42.75	0.65	730	1200	2.78	2	16
684	228	57	0.65	730	1200	3.28	2	15.5
685	310	0	0.6	710	1200	0.4	2	20
686	294.5	15.5	0.6	710	1200	0.88	2	20
687	279	31	0.6	710	1200	1.7	2	19.5
688	263.5	46.5	0.6	710	1200	2.74	2	19
689	248	62	0.6	710	1200	3.22	2	19

690	355	0	0.52	670	1200	0.4	2	26
691	337.25	17.75	0.52	670	1200	1.85	2	27
692	319.5	35.5	0.52	670	1200	3.35	2	26.5
693	301.75	53.25	0.52	670	1200	4.16	2	26.5
694	284	71	0.52	670	1200	4.14	2	26
695	410	0	0.45	625	1200	0.86	2	32
696	389.5	20.5	0.45	625	1200	3.27	2	34
697	369	41	0.45	625	1200	4.35	2	33.5
698	348.5	61.5	0.45	625	1200	4.67	2	33.5
699	328	82	0.45	625	1200	4.95	2	33.5
700	385	0	0.46	911	214	0.96	2	12.14
701	513	27	0.3	859	825	3.24	2	27.52
702	496.8	43.2	0.3	856	822	3.24	2	29.14
703	480.6	59.4	0.3	853	820	3.24	2	30.81
704	450	0	0.28	675	1125	14	1	43.2
705	427.5	22.5	0.28	675	1125	14	1	47.3
706	405	45	0.28	675	1125	14	1	46.9

707	382.5	67.5	0.28	675	1125	14	1	46.4
708	400	0	0.45	793	1000	1.3	7	41.4
709	390	10	0.45	790.7	1000	1.45	7	43.2
710	380	20	0.45	788.4	1000	1.45	7	43.2
711	360	40	0.45	783.8	1000	1.6	7	47.7
712	400	0	0.45	793	1000	1.3	28	46.8
713	390	10	0.45	790.7	1000	1.45	28	53.1
714	380	20	0.45	788.4	1000	1.45	28	54
715	360	40	0.45	783.8	1000	1.6	28	59.4
716	400	0	0.45	793	1000	1.3	56	49.5
717	390	10	0.45	790.7	1000	1.45	56	58.5
718	380	20	0.45	788.4	1000	1.45	56	59.4
719	360	40	0.45	783.8	1000	1.6	56	61.2
720	400	0	0.45	793	1000	1.3	91	54
721	390	10	0.45	790.7	1000	1.45	91	61.2

722	380	20	0.45	788.4	1000	1.45	91	63.9
723	360	40	0.45	783.8	1000	1.6	91	66.6
724	400	0	0.45	793	1000	1.3	180	55.8
725	390	10	0.45	790.7	1000	1.45	180	63.9
726	380	20	0.45	788.4	1000	1.45	180	65.7
727	360	40	0.45	783.8	1000	1.6	180	69.3
728	360	72	0.42	1109	612	0	7	20.35
729	360	72	0.42	1109	612	0	28	20.9
730	405	40.5	0.42	1109	612	0	7	22.165
731	495	49.5	0.42	909	612	0	7	24.75
732	405	40.5	0.42	1109	612	0	28	24.75
733	440	88	0.42	909	612	0	7	25.85
734	440	88	0.42	909	612	0	28	27.335
735	382.5	57.4	0.42	1109	612	0	7	29.7
736	520	0	0.37	890	900	7.8	1	32.34

737	467.5	70.1	0.42	909	612	0	7	33
738	364	156	0.37	890	900	7.8	1	33.11
739	390	130	0.37	890	900	7.8	1	33.44
740	416	104	0.37	890	900	7.8	1	33.66
741	382.5	57.4	0.42	1109	612	0	28	33.88
742	495	49.5	0.42	909	612	0	28	34.1
743	442	78	0.37	890	900	7.8	1	34.43
744	468	52	0.37	890	900	7.8	1	35.64
745	400	0	0.38	835	1047	4	28	35.64
746	520	0	0.37	890	900	7.8	7	36.63
747	494	26	0.37	890	900	7.8	1	38.17
748	467.5	70.1	0.42	909	612	0	28	38.225
749	350	0	0.5	1315	576	0.096	28	39.38
750	400	20	0.36	835	1047	4	28	40.04
751	332.5	17.5	0.5	1315	576	0.289	28	43.89
752	494	26	0.37	890	900	7.8	7	49.06

753	468	52	0.37	890	900	7.8	7	49.72
754	315	35	0.5	1315	576	0.501	28	50.05
755	389.5	19.5	0.5	605	1132	0	28	51.48
756	416	22	0.4	691	1088	7.66	28	51.535
757	442	78	0.37	890	900	7.8	7	52.58
758	416	104	0.37	890	900	7.8	7	54.12
759	390	130	0.37	890	900	7.8	7	54.23
760	364	156	0.37	890	900	7.8	7	54.56
761	520	0	0.37	890	900	7.8	28	55.88
762	450	0	0.45	775	719	5.4	400	56.54
763	394.2	43.8	0.4	691	1088	7.66	28	57.816
764	461.7	24.3	0.35	664	1088	9.72	28	57.959
765	389.5	19.5	0.5	605	1132	0	56	59.62
766	437.4	48.6	0.35	664	1088	9.72	28	54.733
767	400	40	0.34	835	1047	4	28	55.076
768	400	60	0.33	835	1047	4	28	58.898
769	522.5	27.5	0.3	624	1088	13.75	28	58.898
770	405	45	0.45	775	719	5.4	400	58.9176

771	494	26	0.37	890	900	7.8	28	59.976
772	450	0	0.4	771	744	5.4	400	60.6032
773	468	52	0.37	890	900	7.8	28	60.858
774	495	55	0.3	624	1088	13.75	28	62.5828
775	405	45	0.4	744	744	5.4	400	63.161
776	380	19	0.4	688	1157	5.5	28	63.504
777	380	19	0.4	688	1157	5.5	56	63.504
778	442	78	0.37	890	900	7.8	28	64.974
779	450	0	0.35	798	770	5.4	400	65.1308
780	416	104	0.37	890	900	7.8	28	68.698
781	608	32	0.25	562	1088	17.6	28	70.2072
782	405	45	0.35	798	770	5.4	400	70.756
783	576	64	0.25	562	1088	17.6	28	73.3726
784	450	0	0.25	905.36	979.66	18	28	73.402
785	478.4	41.6	0.31	805.1	914.3	3.38	28	73.99
786	390	130	0.37	890	900	7.8	28	77.616
787	468	52	0.31	805.1	914.3	3.38	28	79.478

788	364	156	0.37	890	900	7.8	28	81.242
789	457.6	62.4	0.31	805.1	914.3	3.38	28	81.34
790	337.5	112.5	0.25	889.81	962.83	13.5	28	83.1824
791	475	23.75	0.3	719	1086	8	28	85.946
792	427.5	22.5	0.25	902.24	976.29	18	28	89.3368
793	475	23.75	0.3	719	1086	8	56	90.454
794	405	45	0.25	899.13	972.93	15.75	28	94.3642
795	360	90	0.25	892.91	966.2	13.5	28	96.4908
796	382.5	67.5	0.25	896.03	969.56	15.75	28	101.871

Table S2. Experimental database of splitting tensile strength of concrete incorporating silica fume.

S.No.	Cement (kg/m^3)	Silica fume (kg/m^3)	w/b	Fine aggregate (kg/m^3)	Coarse aggregate (kg/m^3)	SP (kg/m^3)	Days	Splitting tensile strength (MPa)
1	475	23.75	0.3	719	1086	8	28	5.24
2	380	19	0.4	688	1157	5.5	28	4.16
3	389.5	19.47	0.5	605	1132	0	28	3.6
4	475	23.75	0.3	719	1086	8	56	5.33
5	380	19	0.4	688	1157	5.5	56	4.61
6	389.5	19.47	0.5	605	1132	0	56	3.68
7	427.5	22.5	0.36	990	735	8	28	4.36

8	405	45	0.38	990	735	8	28	4.99
9	382.5	67.5	0.4	990	735	8	28	4.87
10	360	90	0.4	990	735	8	28	4.7
11	478.4	41.6	0.31	805.1	914.3	3.38	28	6.07
12	468	52	0.31	805.1	914.3	3.38	28	6.64
13	457.6	62.4	0.31	805.1	914.3	3.38	28	6.47
14	600	0	0.3	613.6	1084.6	7.2	3	2.563
15	600	30	0.3	594.69	1051.1	7.46	3	2.331
16	600	60	0.3	585.55	1017.7	7.8	3	2.868
17	600	90	0.3	556.85	984.3	8.13	3	2.844
18	600	0	0.3	613.6	1084.6	7.2	7	3.215
19	600	30	0.3	594.69	1051.1	7.46	7	3.906
20	600	60	0.3	585.55	1017.7	7.8	7	3.617
21	600	90	0.3	556.85	984.3	8.13	7	3.199
22	600	0	0.3	613.6	1084.6	7.2	28	4.57
23	600	30	0.3	594.69	1051.1	7.46	28	4.6
24	600	60	0.3	585.55	1017.7	7.8	28	3.939
25	600	90	0.3	556.85	984.3	8.13	28	3.578
26	400	0	0.38	835	1047	4	28	3.48
27	400	20	0.36	835	1047	4	28	3.82
28	400	40	0.34	835	1047	4	28	5.36
29	400	60	0.33	835	1047	4	28	6.54
30	385	0	0.46	920	884	2.31	7	2.4297
31	354.2	30.8	0.46	915	878	2.7	7	2.6845
32	450	0	0.36	912	877	4.95	7	3.2396

33	414	36	0.36	906	870	5.4	7	3.6218
34	385	0	0.46	920	884	2.31	28	2.9302
35	354.2	30.8	0.46	915	878	2.7	28	3.2032
36	450	0	0.36	912	877	4.95	28	3.9949
37	414	36	0.36	906	870	5.4	28	4.2861
38	385	0	0.46	920	884	2.31	91	3.5399
39	354.2	30.8	0.46	915	878	2.7	91	3.6127
40	450	0	0.36	912	877	4.95	91	4.3134
41	414	36	0.36	906	870	5.4	91	5.0232
42	416	22	0.4	691	1088	7.66	28	3.88
43	394.2	43<br>.8	0.4	691	1088	7.66	28	4.38
44	461.7	24.3	0.35	664	1088	9.72	28	4.41
45	437.4	48.6	0.35	664	1088	9.72	28	4.75
46	522.5	27.5	0.3	624	1088	13.75	28	4.86
47	495	55	0.3	624	1088	13.75	28	5.12
48	608	32	0.25	562	1088	17.6	28	5.15
49	576	64	0.25	562	1088	17.6	28	5.62
50	450	0	0.44	599	1148	0	28	3.01
51	432	18	0.44	599	1148	0	28	3.36
52	414	36	0.44	599	1148	0	28	3.43
53	396	54	0.44	599	1148	0	28	3.17
54	315	215	0.45	960	813	6.2	7	3.07
55	357	196	0.4	960	813	6.3	7	4.87
56	411	135	0.35	960	813	7.29	7	5.86

57	315	215	0.45	960	813	6.2	28	4.3
58	357	196	0.4	960	813	6.3	28	5.58
59	411	135	0.35	960	813	7.29	28	6.72
60	450	0	0.4	688	1062	13.5	90	4.1
61	428	22.5	0.4	685	1058	13.5	90	4.3
62	405	45	0.4	682	1054	13.5	90	4.5
63	383	67.5	0.4	680	1049	13.5	90	4.7
64	360	90	0.4	677	1045	13.5	90	4.7
65	350	0	0.6	697	1076	5.3	90	3.1
66	333	17.5	0.6	695	1073	5.3	90	3.18
67	315	35	0.6	693	1070	5.3	90	3.28
68	298	52.5	0.6	691	1066	5.3	90	3.7
69	280	70	0.6	688	1063	5.3	90	3.7
70	350	0	0.5	1315	576	0.096	28	2.25
71	332.5	17.5	0.5	1315	576	0.289	28	2.38
72	315	35	0.5	1315	576	0.501	28	3.09
73	378.85	0	0.52	718.48	1224.97	0	7	1.29
74	359.95	18.9	0.52	718.48	1224.97	0	7	1.41
75	340.97	37.88	0.52	718.48	1224.97	0	7	1.69
76	322.05	56.8	0.52	718.48	1224.97	0	7	1.27
77	303.08	75.77	0.52	718.48	1224.97	0	7	1.1
78	378.85	0	0.52	718.48	1224.97	0	28	2.54
79	359.95	18.9	0.52	718.48	1224.97	0	28	2.68
80	340.97	37.88	0.52	718.48	1224.97	0	28	3.25
81	322.05	56.8	0.52	718.48	1224.97	0	28	2.47

82	303.08	75.77	0.52	718.48	1224.97	0	28	2.26
83	378.85	0	0.52	718.48	1224.97	0	56	2.75
84	359.95	18.9	0.52	718.48	1224.97	0	56	2.82
85	340.97	37.88	0.52	718.48	1224.97	0	56	3.32
86	322.05	56.8	0.52	718.48	1224.97	0	56	2.44
87	303.08	75.77	0.52	718.48	1224.97	0	56	2.28
88	378.85	0	0.52	718.48	1224.97	0	90	2.96
89	359.95	18.9	0.52	718.48	1224.97	0	90	3.1
90	340.97	37.88	0.52	718.48	1224.97	0	90	3.55
91	322.05	56.8	0.52	718.48	1224.97	0	90	2.8
92	303.08	75.77	0.52	718.48	1224.97	0	90	2.49
93	520	0	0.3	860	886	5.2	7	4.2133
94	468	52	0.3	851	877	5.2	7	4.5682
95	520	0	0.3	860	886	5.2	28	4.7957
96	468	52	0.3	851	877	5.2	28	5.3508
97	520	0	0.3	860	886	5.2	91	5.2871
98	468	52	0.3	851	877	5.2	91	5.733
99	500	0	0.35	900	600	17.5	7	1.08
100	350	150	0.35	900	600	10.5	7	1.84
101	300	200	0.35	900	600	10.35	7	1.61
102	250	250	0.35	900	600	10	7	1.49
103	500	0	0.35	900	600	17.5	28	1.74
104	350	150	0.35	900	600	10.5	28	2.14
105	300	200	0.35	900	600	10.35	28	1.9
106	250	250	0.35	900	600	10	28	1.51

107	700	0	0.18	1230	0	70	7	3.6127
108	700	105	0.156	1230	0	70	7	5.3781
109	700	210	0.14	1230	0	70	7	6.9069
110	750	0	0.18	1230	0	75	7	4.9322
111	750	112.5	0.156	1230	0	75	7	7.5985
112	750	225	0.14	1230	0	75	7	8.5267
113	800	0	0.18	1230	0	80	7	7.1253
114	800	120	0.156	1230	0	80	7	8.372
115	800	240	0.14	1230	0	80	7	9.8917
116	700	0	0.18	1230	0	70	28	4.5318
117	700	105	0.156	1230	0	70	28	5.5419
118	700	210	0.14	1230	0	70	28	7.0252
119	750	0	0.18	1230	0	75	28	5.5328
120	750	112.5	0.156	1230	0	75	28	7.7896
121	750	225	0.14	1230	0	75	28	8.645
122	800	0	0.18	1230	0	80	28	7.3892
123	800	120	0.156	1230	0	80	28	8.5904
124	800	240	0.14	1230	0	80	28	10.0009
125	600	0	0.33	900	750	12	28	3.5

126	570	30	0.33	900	750	12	28	4.6
127	540	60	0.32	900	750	12	28	4.37
128	450	0	0.4	670	1100	1.125	7	3.59
129	405	45	0.4	670	1100	4.5	7	4.36
130	450	0	0.4	670	1100	1.125	28	4.19
131	405	45	0.4	670	1100	4.5	28	5.2
132	450	0	0.4	670	1100	1.125	56	4.39
133	405	45	0.4	670	1100	4.5	56	5.21
134	450	0	0.4	670	1100	1.125	90	5.15
135	405	45	0.4	670	1100	4.5	90	5.63
136	495	24.75	0.3	535	1248	5.13	7	5.02
137	470	24.75	0.3	535	1248	5.13	7	5.62
138	495	24.75	0.3	535	1248	5.13	28	7.36
139	470	24.75	0.3	535	1248	5.13	28	7.92
140	495	24.75	0.3	535	1248	5.13	56	8.03
141	470	24.75	0.3	535	1248	5.13	56	8.45
142	610	50	0.22	631	1070	7.75	1	3.91
143	500	50	0.31	656	1070	3.25	1	2.48
144	286	32	0.55	843	1070	0	1	0.87
145	197	21	0.83	916	1070	0	1	0.51
146	610	50	0.22	631	1070	7.75	3	4.86
147	500	50	0.31	656	1070	3.25	3	4.33
148	286	32	0.55	843	1070	0	3	2.48
149	197	21	0.83	916	1070	0	3	1.44
150	610	50	0.22	631	1070	7.75	7	5.48

151	500	50	0.31	656	1070	3.25	7	5.22
152	286	32	0.55	843	1070	0	7	2.87
153	197	21	0.83	916	1070	0	7	1.78
154	610	50	0.22	631	1070	7.75	28	6.83
155	500	50	0.31	656	1070	3.25	28	5.98
156	286	32	0.55	843	1070	0	28	3.74
157	197	21	0.83	916	1070	0	28	2.82
